# Leveraging a national biorepository in Zambia to assess measles and rubella immunity gaps across age and space

**DOI:** 10.1038/s41598-022-14493-3

**Published:** 2022-06-17

**Authors:** Andrea C. Carcelen, Amy K. Winter, William J. Moss, Innocent Chilumba, Irene Mutale, Gershom Chongwe, Mwaka Monze, Gina Mulundu, Hope Nkamba, Francis. D. Mwansa, Lloyd Mulenga, Dale A. Rhoda, Kyla Hayford, Simon Mutembo

**Affiliations:** 1 International Vaccine Access Center, Department of International Health, Johns Hopkins Bloomberg School of Public Health, 415 N Washington Street, 5th floor, Baltimore, MD 21231 USA; 2 Department of Epidemiology and Biostatistics, University of Georgia, Athens, GA 30602 USA; 3 Department of Epidemiology, Johns Hopkins Bloomberg School of Public Health, Baltimore, MD 21231 USA; 4 W Harry Feinstone Department of Molecular Microbiology and Immunology, Johns Hopkins Bloomberg School of Public Health, Baltimore, MD 21231 USA; 5 Tropical Diseases Research Center, Ndola, Zambia; 6 Department of Pathology and Microbiology, University Teaching Hospital, Lusaka, Zambia; 7 Government of the Republic of Zambia, Ministry of Health, Lusaka, Zambia; 8 Ministry of Health, Directorate of Clinical Care and Diagnostic Service, Government of the Republic of Zambia, Lusaka, Zambia; 9 Biostat Global Consulting, Worthington, OH 43085 USA

**Keywords:** Infectious diseases, Infectious diseases, Vaccines

## Abstract

High-quality, representative serological surveys allow direct estimates of immunity profiles to inform vaccination strategies but can be costly and logistically challenging. Leveraging residual serum samples is one way to increase their feasibility. We subsampled 9854 residual sera from a 2016 national HIV survey in Zambia and tested these specimens for anti-measles and anti-rubella virus IgG antibodies using indirect enzyme immunoassays. We demonstrate innovative methods for sampling residual sera and analyzing seroprevalence data, as well as the value of seroprevalence estimates to understand and control measles and rubella. National measles and rubella seroprevalence for individuals younger than 50 years was 82.8% (95% CI 81.6, 83.9%) and 74.9% (95% CI 73.7, 76.0%), respectively. Despite a successful childhood vaccination program, measles immunity gaps persisted across age groups and districts, indicating the need for additional activities to complement routine immunization. Prior to vaccine introduction, we estimated a rubella burden of 96 congenital rubella syndrome cases per 100,000 live births. Residual samples from large-scale surveys can reduce the cost and challenges of conducting serosurveys, and multiple pathogens can be tested. Procedures to access quality specimens, ensure ethical approvals, and link sociodemographic data can improve the timeliness and value of results.

## Introduction

Improvement in vaccination coverage has substantially reduced the global burden of measles and rubella. However, progress toward measles elimination has slowed or reversed since 2016 due to the challenges of achieving and sustaining population immunity above the 92–95% threshold needed to interrupt transmission^1^. Disruptions to vaccination programs caused by the COVID-19 pandemic have further impeded control and elimination efforts in some countries^2^.

The World Health Organization (WHO) recommends countries use high quality vaccination coverage and case data to monitor population immunity to measles and identify remaining susceptible populations requiring vaccination^3,4^. However, profiles of immunity across age and space are difficult to reconstruct given the poor quality of administrative vaccination data, infrequent and potentially biased vaccination coverage surveys, and under-reporting of disease case data^5,6^. This has been particularly problematic in countries where coverage appeared to be sufficient in reducing the number of reported measles cases but susceptible individuals accumulated, resulting in unexpected, large outbreaks^7^. Additionally, these data sources often mask subnational geographic variability and clustering of unvaccinated individuals that become increasingly important to identify as countries get closer to elimination^8,9^. Disruptions to health services like the COVID-19 pandemic further complicate reconstruction of immunity profiles because data on vaccination coverage and disease surveillance become less complete^2^.

High-quality representative serological surveys provide a more accurate assessment of immunity profiles^6,10^. Serosurveys can be used to estimate key epidemiological parameters and disease burden, assess outbreak risk, and evaluate and guide immunization programs^10–13^. However, there are many challenges to conducting, analyzing, and interpreting serosurveys that hinder their use, particularly in low and middle income countries^10,14,15^. It is important to explore approaches to increase the feasibility of conducting serosurveys.

We demonstrate one approach to increase the feasibility of conducting serosurveys and the value of these data to estimate measles and rubella immunity gaps and inform elimination strategies in Zambia. We leveraged a provincially representative biorepository of blood samples collected in 2016 as part of the Zambian Population HIV Impact Assessment (ZAMPHIA) study to conduct a nested serosurvey to estimate population immunity to measles and rubella for all ten provinces in Zambia. There are broad implications of this work given that blood specimens are routinely collected (e.g., HIV impact assessments, demographic health surveys (DHS), multiple indicator cluster surveys (MICS), and current SARS-CoV-2 seroprevalence studies) that could be accessed to estimate population immunity to multiple pathogens, including measles and rubella.

## Results

### Measles

**Figure 1:**
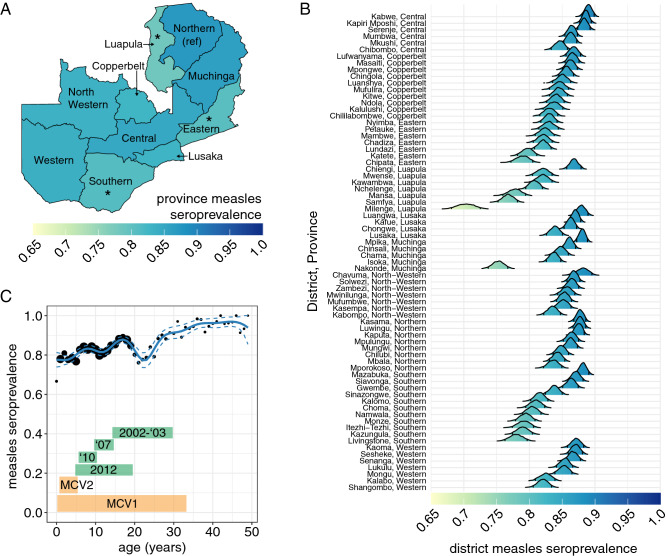
Measles seroprevalence. (**A**) Province seroprevalence. The asterisks represent provinces that had significantly different seroprevalence than the reference province, Northern Province (0.05 alpha level). (**B**) District seroprevalence. The distribution of district-specific seroprevalence estimates based on a hierarchical spatial model. The left and right vertical lines within each distribution represent the 5th and 95th percentiles. (**C**) National age-specific seroprevalence. The points represent the data grouped by age in years with the exception of year 0 which includes only individuals 10 and 11 months old and the size of the point is proportional to the number of observations in each age group (seroprevalence estimates for individuals younger than 10 months were collected and are show in Fig. S8). The blue lines represent fitted generalized additive model mean (solid line) and 95% confidence intervals (dashed lines). The age cohorts eligible for vaccination campaigns by campaign year (green boxes) and routine doses of measles-containing vaccine dose 1 (MCV1) and dose 2 (MCV2) (orange boxes) are shown.

The national measles seroprevalence for individuals younger than 50 years was 82.8% (95% CI 81.6, 83.9%). Measles seroprevalence ranged across provinces from 78.2% (95% CI 75.2%, 81.4%) in Luapula to 87.0% (95% CI 84.3%, 89.7%) in Northern, with statistically significant differences between them (Fig. 1A, Table S1). District-specific seroprevalence estimates revealed variation within and across districts (Fig. 1B). Milenge District in Luapula Province and Kabwe District in Central Province had the lowest and highest estimated mean seroprevalence (70.3% and 89.0%), respectively. Mean seroprevalence was greater than 85% in about half of the districts (34 of 72, 47%) but no district-specific seroprevalence surpassed 90%.

**Figure 2:**
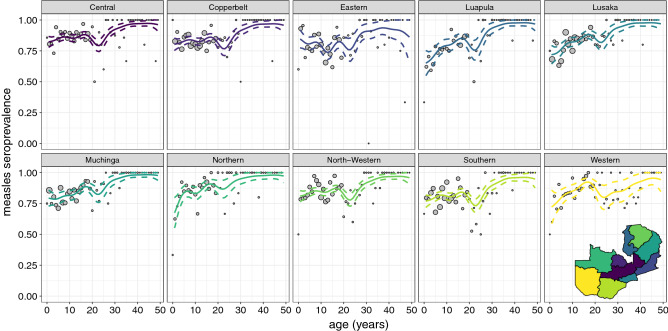
Measles age-specific seroprevalence by province. The points represent the data grouped by age in years with the exception of year 0 which includes only individuals 10 and 11 months old and are proportional to the number of individuals in each age in years. The lines represent the generalized additive model mean (solid line) and 95% confidence intervals (dashed lines).

Measles seroprevalence increased with age (Fig. 1C). Estimated seroprevalence by age group was 71% among children 0–4 years old, 82% for age groups 5–9 and 10–14 years old, and 87% and 88% for individuals 15–19 and 20–49 years old (Table S2, Fig. S1). Males had significantly lower measles seroprevalence (81%; 95% CI 80%, 83%) than females (84%; 95% CI 82%, 86%; Table S3), specifically among individuals younger than 20 years (Fig. S2). Fitting a generalized additive model to seropositivity by age, measles seroprevalence was slightly lower in the 20–25 year age group (Fig. 1C). A univariate binomial model confirmed measles seroprevalence was lower among those 20–25 years of age compared to those 15–19 and 26–49 years of age. This decrease in seroprevalence included birth cohorts who had two opportunities for vaccination, including routine vaccination and a vaccination campaign conducted in 2002–2003 (Fig. 1C). Lower measles seroprevalence among those 20–25 years old was seen in all provinces, although the magnitude of this decrease in seroprevalence varied (Fig. 2), and age-group seroprevalence estimates differed across provinces (Table S4 and Fig S1). Natural immunity would be most likely in age cohorts older than 14 years in 2016 as relatively high measles incidence was reported prior to 2002 (Fig. S3).

**Figure 3:**
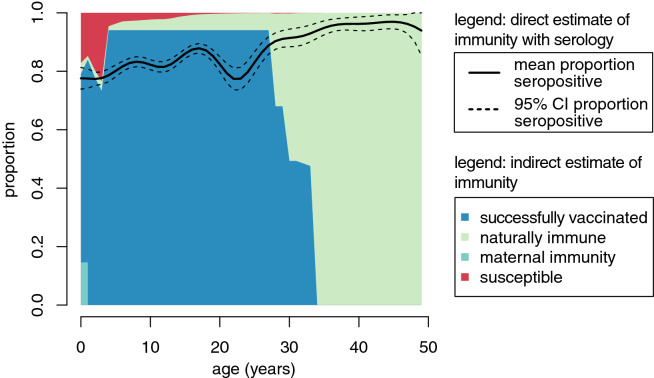
Comparison of two estimates of national age-specific measles immunity profiles. The direct estimate, based on generalized additive models fit to the seroprevalence data, is represented as the solid (mean) and dashed (95% CI) lines. The indirect estimate is based on a birth-cohort method that estimates immunity per birth cohort. Each birth cohort has a unique probability of being successfully vaccinated (dark blue), a probability of being infected (light green), and among infants a probability of being immune through maternally-acquired antibodies (teal). The proportion of the population each age that remains susceptible is shown in red.

A reconstructed national age-specific immunity profile using the indirect birth cohort method predicted a higher proportion immune in most age groups than national age-specific seroprevalence estimates and did not provide an explanation for the decrease in seroprevalence among those 20–25 years of age in our sample (Fig. 3). The differences were larger for individuals eligible for vaccination campaigns (four years and older) and increased over age (Fig. 3). There was good agreement between reconstructed immunity and seroprevalence estimates among individuals younger than four years (Fig. 3). This finding held true at the provincial level in which administrative routine vaccination data were informative of seroprevalence for individuals younger than three years, particularly when the denominator used to estimate vaccination coverage was based on provincial-specific birth rates rather than a national birth rate (Fig. S4); although it was no better at estimating provincial rank by seroprevalence (Fig. S5).

At the time of serological data collection, there was high risk for a measles outbreak in Zambia. National seroprevalence of 78.3% (95% CI 75.2%, 81.4%) was below the measles critical immunity threshold (92–95%) and estimated R_eff_ was above the unity threshold (mean 2.21; 95% CI 2.00, 2.44). In the following year (2017), we estimated that seroprevalence increased and R_eff_ decreased closer to one (1.21) as a result of the MR vaccination campaign in late 2016 targeting ages 9 months to 14 years. The estimated risk of measles outbreaks subsequently increased as susceptible children accumulated, with a mean R_eff_ in 2018 and 2019 of 1.25 and 1.31, respectively.

### Rubella

**Figure 4:**
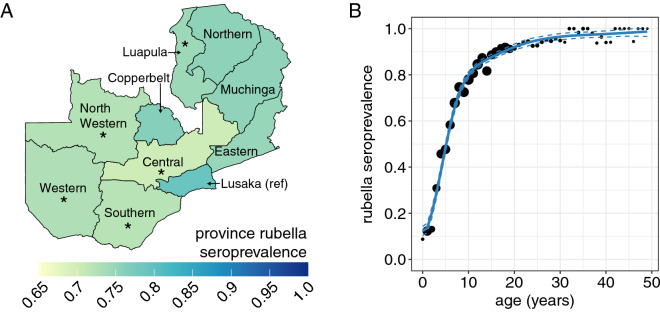
Rubella seroprevalence. (**A**) Province seroprevalence. The asterisks represent provinces that had significantly different seroprevalence than the reference province, Lusaka Province (0.05 alpha level). (**B**) National age-specific seroprevalence. The points represent the data grouped by age in years with the exception of year 0 which includes only individuals 10 and 11 months old and the size of the point is proportional to the number of observations in each age group (seroprevalence estimates for individuals younger than 10 months was collected and is show in Fig. S8). The blue lines represent fitted generalized additive model mean (solid line) and 95% confidence intervals (dashed lines). Rubella vaccine was not available in the public sector prior to the serosurvey.

The national rubella seroprevalence for individuals younger than 50 years was 74.9% (95% CI 73.7%, 76.0%). Rubella seroprevalence ranged across provinces, from 70.1% (95% CI 66.7%, 73.5%) in Central Province to 78.9% (95% CI 75.9%, 82.1%) in Lusaka Province (Fig. 4A, Table S1). Compared to Lusaka Province, where the capital city is located, five of the other nine provinces had significantly lower rubella seroprevalence (Fig. 4A).

**Figure 5:**
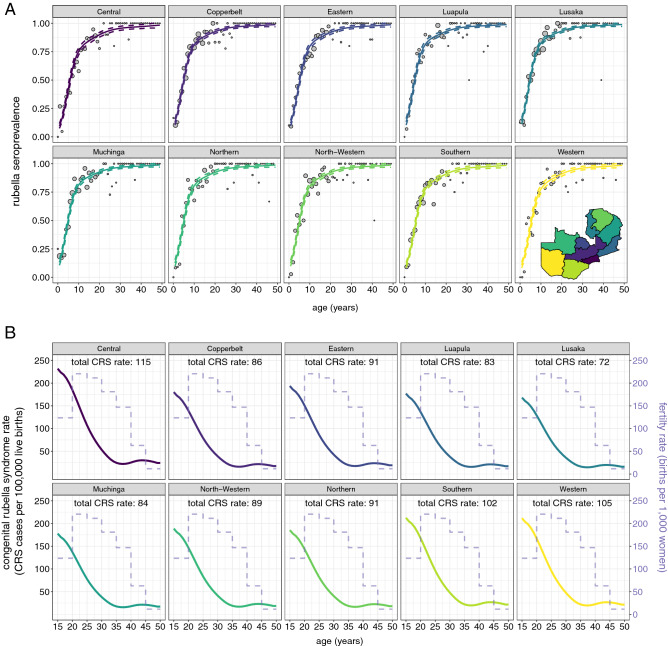
Age-specific rubella seroprevalence and CRS rate by province. (**A**) Rubella age-specific seroprevalence by province. The points represent the data grouped by age in years with the exception of year 0 which includes only individuals 10 and 11 months old and are proportional to the number of individuals in each age in years. The lines represent fitted generalized additive model mean (solid line) and 95% confidence intervals (dashed lines). (**B**) Provincial age-specific CRS rate (solid line), and national age-specific fertility rate (dashed line). The total provincial CRS rates (labeled in text at the top of each plot) is the weighted average of the age-specific CRS rate by age-specific fertility rate.

Rubella seroprevalence increased steadily with age, consistent with cumulative exposure to rubella virus in endemic settings (Fig. 4B). Estimated seroprevalence by age group was 27.3%, 65.1%, 82.3%, 91.2%, and 95.7% among individuals 0–4, 5–9, 10–14, 15–19 and 20–49 years, respectively (Table S2). There was no significant difference in rubella seroprevalence by sex (Table S3). Fitting a generalized additive model to rubella seropositivity by age, rubella seroprevalence increased after 9 months of age when maternal immunity has waned^16^ but eventually plateaued as the number of remaining susceptible individuals declined (Fig. 5A). While the overall shape of the rubella age-specific seroprevalence curves were similar by province, small differences in the force of infection were identified (Fig. S6), resulting in differences in the magnitude of seroprevalence by age (Fig. 5A, Fig. S7, Table S4).

The burden of rubella is characterized by CRS rate. Provinces with a lower force of infection among children will have more remaining susceptible individuals of reproductive age who, if pregnant, will be at a higher risk of having infants affected by CRS. Figure 5B displays the estimated age-specific and total CRS rates by province. The estimated annual number of CRS cases was 76, 77, 76, 45, 82, 37, 54, 34, 82, and 42 in Central, Copperbelt, Eastern, Luapula, Luska, Muchinga, Northern, North-Western, Southern, and Western Provinces, respectively. Central Province, with the lowest force of infection up to 15 years old (Fig. S6), had the highest age-specific rate of CRS (Fig. 5B) and total CRS rate (115 CRS cases per 100,000 live births); however, Central Province did not have the most CRS cases because the number of births in this province was fewer than other provinces. Aggregating the provincial estimates, 605 CRS cases were estimated to occur annually in Zambia, equivalent to 96 CRS cases per 100,000 live births. The estimated rubella basic reproductive number, i.e., the average number of infected contacts per a typical infectious individual in a completely susceptible population, was 3.03 (95% CI 2.42, 4.01).

## Discussion

By accessing a nationally representative biorepository, we generated estimates of measles and rubella seroprevalence over different spatial scales and age groups to identify immunity gaps that can be used to target vaccination strategies. Biorepositories provide the opportunity to conduct serological surveillance without the burden of specimen collection and allow for serological testing for multiple pathogens using the same specimens^17^. Biorepositories may be readily available in many low and middle-income countries, such as residual blood bank specimens, antenatal care specimens, or specimens from other surveys (e.g., DHS, MICS), that could be accessed for serological surveys. However, several factors should be considered when selecting a biorepository, including logistical issues such as appropriate ethical approvals, how the specimens were originally collected and stored, and if demographic data can be linked to specimens, as well as potential for biases resulting from time since specimen collection, potential temporal trends in seroprevalence, and whether the biorepository is representative of the target population^15,18,19^. The frequency of recently conducted serosurveys to characterize the SARS-CoV-2 pandemic provides an additional opportunity to leverage biorepositories to probe the epidemiology of other vaccine-preventable, endemic, and emerging infectious diseases.

Measles seroprevalence estimates provided evidence of an effective vaccination program, with seroprevalence ranging 71–88% among children born since the last large national outbreak (2010–2011). However, measles seroprevalence remained lower than the 92–95% critical immunity threshold, and immunity gaps in some age groups and many districts were identified. Despite an outbreak risk, there were no national measles outbreaks following the serosurvey, likely because a mass measles-rubella vaccination campaign was conducted six months later in 2016, reducing R_eff_. The vaccination campaign was conducted in every district which, based on our district level estimates, was appropriate because population seroprevalence was lower than 90% in all districts. In the years following the 2016 vaccination campaign, the risk of measles outbreak increased and remained high. To reach measles elimination in Zambia, routine vaccination must more effectively immunize susceptible individuals if the country is to decrease reliance on mass vaccination campaigns. Potential strategies include increasing low MCV2 coverage (Fig. S3).

Measles seroprevalence estimates were lower than reconstructed immunity estimates that did not include serological data, specifically for birth cohorts eligible for vaccination campaigns and at higher risk for natural infection. However, there was reasonable agreement between reconstructed immunity and the seroprevalence estimates for children younger than four years at national and provincial levels. Routine administrative coverage data may be informative of seroprevalence in young children in the absence of opportunities for vaccination in campaigns or exposure to wild-type measles virus. We hypothesize this is because reconstructing immunity profiles in the context of campaigns or natural infection is complicated by unknown parameters including the correlation between receiving routine and campaign vaccine doses, true size of the campaign target population, and measles case reporting efficiency.

Our analysis shows that males had slightly, but significantly, lower measles seroprevalence than females, but there was no difference in rubella seroprevalence. In Zambia there are no reported differences in vaccination coverage by sex, although one study in Lusaka suggested boys had more access to vaccination^20,21^. Studies have not found differential antibody responses for measles vaccine by sex^22^, but have demonstrated that females have a higher antibody responses than males to other vaccines^23^. It is not known if these differences in immunologic responses translate into differences in clinical protection.

Reported cases of laboratory-confirmed rubella and CRS 2005–2016^24^ confirm circulating rubella virus^25^. We estimated an annual CRS rate of 96. This estimate is lower but within the 95% confidence intervals of modeled estimates of 157^12^. Six months after the specimens from our analysis were collected, measles-rubella vaccine was introduced through a vaccination campaign targeting children 9 months–14 years and inclusion into the routine vaccination schedule. After this campaign, CRS cases would likely decrease and remain lower than prior to the introduction of rubella vaccine if coverage levels were ≥ 80%^26^. Reported measles-rubella first-dose routine vaccination coverage is 90%, which alone would be sufficiently high for rubella incidence to remain low and provide an opportunity for rubella elimination in Zambia^21^. Measles seroprevalence among children 1 years old was approximately 75%. As a result, the estimated 90% dose one routine vaccination coverage may be over-estimated but not by much; roughly 90% measles vaccine coverage, given 85% measles vaccine effectiveness at 9 months, means ~ 77% of children would be successfully vaccinated or immune to measles. Nonetheless, circulating rubella virus in neighboring countries without rubella vaccine, such as the Democratic Republic of the Congo, continues to pose a risk^27^. Surveillance must improve to rapidly respond to sporadic outbreaks.

This study is subject to the limitations of misclassification and selection bias^14^. EIAs do not measure functional, protective antibodies and risk misclassification particularly for individuals with low antibody levels, who may still have a protective immune response (Supplemental Methods)^15^. Selection bias could have resulted due to the refusal rates among participants in the original ZAMPHIA study, particularly children. Not all specimens selected for testing were available in the biorepository. We accounted for this nonresponse rate in the analyses through weighting. Because we did not have vaccination status for participants, we were not able to estimate associations between vaccination history and seroprevalence. The nested serosurvey was sampled to represent provinces by age-group. We extended the granularity of our estimates using spatial models that account for the data structure; as a result, age-specific and district estimates depend on assumptions inherent in these models^28,29^.

The decrease in measles seroprevalence among 20–25-year-olds could not be fully explained by vaccination data or natural infection. Potential hypotheses are that the decrease in seroprevalence could be due to waning antibodies among individuals who were the earliest birth cohorts to be vaccinated in Zambia^30^. It is also possible that these birth cohorts represent the beginning of the transition from uncontrolled to more controlled measles in Zambia (Figs. S3, S9). Some proportions of these birth cohorts could have been unvaccinated (because lower coverage) and not infected (because vaccine had started to control spread of measles) leaving them susceptible. We find a similar result from a serosurvey from Madagascar which estimated a decrease in seroprevalence among the birth cohorts born soon after measles vaccination increased^11^. However, in Madagascar there was no major outbreak of measles during the lifetime of these birth cohorts, whereas in Zambia, there were two larger outbreaks that occurred in 1999–2002 and 2010–2011 during the birth cohorts of interest lifetime. It remains unclear how these Zambian birth cohorts could have missed infection (or boosting) in the course of these outbreaks^25^.

Epidemiological characteristics assumed in our measles outbreak risk analysis could impact and bias the risk estimates. Assumptions include national vaccination coverage despite subnational heterogeneities^31^ random spatial mixing^32^, and the lack of stochastic processes including introduction from neighboring countries.

In this analysis, we demonstrated the potential of residual sera to increase the feasibility of serosurveys using a nationwide measles and rubella serosurvey in Zambia. Detailed methodology provided by ZAMPHIA partners and reports was critical to the successful interpretation of the data. Although informed consent and laboratory procedures allowed for the creation of a biorepository, challenges in accessing specimens and data resulted in multiple delays. Serosurveys considering using residual sera should ensure policies and procedures for specimen and data access as well as linking sociodemographic and specimen data are clearly established at the outset to allow for the provision of timely results.

We also demonstrated the value of these data to evaluate disease burden, assess vaccination programs, track progress towards elimination, and inform future vaccination strategies. Our analyses show clear spatial and age heterogeneities in seroprevalence. Measles and rubella have relatively straightforward transmission dynamics, thus determinants of heterogeneities are those that primarily impact vaccine and natural infection derived immunity: time and space specific vaccination rates, crude birth rates, contact rates by age and space, and spatial clustering of susceptible populations^6,33^. Serosurveys are summary measures of the complex interplay of these determinants that result in spatial heterogeneities. As a result, seroprevalence estimates at subnational levels can be used to guide the allocation of limited resources, improve vaccination coverage, and increase population immunity in provinces or districts with the most need. In Zambia, overall national and provincial seroprevalence estimates showed some variation. However, district-level seroprevalence estimates demonstrated greater heterogeneity at this smaller spatial scale. We plan to use these data to inform predictive models of district seroprevalence for Zambia beyond 2016. For the next supplemental immunization activity, rather than conduct a nationwide vaccination campaign, Zambia could intensify resources to target districts and focus on reaching children who have not been fully vaccinated.

## Methods

### ZAMPHIA design and study population

From March-August 2016, a cross-sectional household survey with blood collection (ZAMPHIA) was conducted. It was a stratified national multistage cluster survey designed to estimate HIV incidence and the percentage of adults living with HIV with viral load suppression in each province. Participants provided written informed consent, parental permission was obtained children under 18 years old, and assent was obtained for participants 10–17 years old. Up to 14 mL of venous blood (or 1 mL of liquid capillary blood for children younger than 2 years) was collected from adults 15–49 years of age in every selected household and from children younger than 15 years of age in every other household^34^. Specimens were collected from 88.5% of adult respondents 15–49 years old and 68% of children younger than 15 years^34^. Specimens were transported to a central laboratory where whole blood was processed into plasma aliquots and dried blood spot (DBS) samples for storage at − 20 °C. After testing for the primary study was completed, residual plasma and DBS were stored in a biorepository at − 80 °C at the Tropical Diseases Research Center in Ndola, Zambia.

### Nested serosurvey: subsample selection and survey weights

A subsample of residual specimens was selected from the ZAMPHIA biorepository to generate age-specific seroprevalence estimates for measles and rubella in each province with 10% precision for age categories 0–4, 5–9, 10–14, 15–19, and 20–49 years. A total of 11,500 participants were subsampled from the 25,383 ZAMPHIA participants younger than 50 years of age who had blood collected. Specimens were selected based on HIV infection status, geographic cluster, and age to maintain provincial representativeness (Supplemental Methods). Participants older than 50 years of age were not included because they were expected to have high measles and rubella seroprevalence^35^.

Challenges linking sociodemographic data from the ZAMPHIA survey to specimens caused delays and difficulty in specimen selection. Not all the selected specimens could be tested, not all participants provided ethical consent for future testing, and some specimens had insufficient volume or could not be located. We calculated survey weights for each specimen starting with the original ZAMPHIA survey weights and then accounted for the inverse probability of selection at each stage of subsampling, non-availability of specimens, and post-stratification adjustments to represent the provincial-level age and sex structure (Supplemental Methods).

This study was conducted in accordance with relevant guidelines and regulations. Ethical approvals for protocols were provided by Johns Hopkins Bloomberg School of Public Health as well as the Tropical Disease Research Center and the National Health Regulatory Agency in Zambia.

### Specimen testing

Plasma specimens were thawed overnight at 4 °C, processed as recommended by the manufacturer, and tested for anti-measles virus IgG and anti-rubella virus IgG antibodies using indirect enzyme immunoassays (EIA; Euroimmun; Lübeck, Germany). For DBS, a 6 mm punch was eluted in 450 µL of sample buffer. The DBS elution protocol was optimized for use with the Euroimmun assays and validated on a subset of paired plasma and DBS. A quantitative antibody concentration was generated using the plate-specific standard curve from plotting the four calibrators. Samples were classified as positive, negative, or equivocal according to the manufacturer thresholds. Measles results were classified as positive (≥ 200 mIU/mL), equivocal (≥ 150 to < 200 mIU/mL), or negative (< 150 mIU/mL). Similarly, rubella results were classified as positive (≥ 11 IU/mL), equivocal (≥ 8 to < 11 IU/mL), or negative (< 8 IU/mL). Specimens with equivocal results were retested. Retest results were used but, if equivocal again, were categorized as positive for the primary analysis. Testing was conducted at the National Virology Laboratory at the University Teaching Hospital in Lusaka and the Tropical Disease Research Center in Ndola between April and October 2019. Internal controls and sample panels were tested, and near-real time data management was conducted to monitor variability.

### Analysis and modeling

We used the R package *survey* to account for the complex survey design in national, provincial, and age-group measles and rubella seroprevalence estimates^36^. Univariate and multivariate generalized linear regression models were fit to evaluate associations between seropositivity and categorical variables (province, age group, sex), taking into account survey weights. All 95% confidence intervals were calculated using linearized Taylor series variance estimation. T-tests were relied upon to determine if there was a significant difference between survey weighted mean seroprevalence between groups.

For national and provincial age-specific measles and rubella seroprevalence, a hierarchical generalized additive model was fit to individual seropositivity. The models included a single national level smoother over age. Final models were selected by minimizing the Akaike Information Criterion (AIC). We reconstructed the national age-profile of measles immunity using vaccination and case data. This indirect method estimates the proportion of each birth cohort that is immune based on vaccination coverage (per routine^37^ and campaigns^38^), age-specific vaccine effectiveness (85% at 9 months, 95% at 12 months), natural infection (per annual force of infection^39^), and maternally derived immunity^40^.

To estimate district-specific measles seroprevalence, a hierarchical spatial model was fit to individual seropositivity. District-specific random effects were included in the model based on a conditional autoregressive (CAR) specification where adjacent districts were assumed to be more similar than non-adjacent districts. We explored model covariates of routinely collected data (e.g., individual HIV positivity, district routine vaccination coverage) and demographic data (e.g., age, population density). The final model was selected by minimizing the Widely Applicable Information Criterion (WAIC).

We estimated national measles outbreak risk using the 2016 cross-sectional serological data to predict seroprevalence for 2017–2019 based on methods developed by Funk et al.^41^. We focused on changes in immunity due to vaccination, assuming no immunity from natural infection given the small number of measles cases reported since 2016 (average of 11 annual cases between 2016 and 2019)^25^. Using age-specific seroprevalence and an assumed who-acquires-infection-from-whom matrix (WAIFW), we estimated the measles effective reproduction number (R_eff,_ average number of secondary cases per infectious individual) 2016–2019. If R_eff_ > 1, cases increase and there is risk of an outbreak. If R_eff_ < 1, cases decline, and transmission will eventually cease.

Because sera were collected before the introduction of rubella-containing vaccine, we evaluated the rubella basic reproductive number and burden of congenital rubella syndrome (CRS) prior to vaccination. The estimated CRS rate (CRS incident cases per 100,000 live births) for each reproductive age in years and province was calculated by $(1-{\pi }_{a,p}) \times (1-{exp}^{-16{\lambda }_{a,p}/52}) \times 0.65 \times \mathrm{100,000}$, where ${\pi }_{a,p}$ is the estimated seroprevalence at age *a* and province *p*, ${\lambda }_{a,p}$ is the estimated force of infection at age *a* and province *p*. We assumed 65% of infants born to women infected during the first 16 weeks of pregnancy were born with CRS^12^.

All computations were done in R, version 4.0.5^42^. Supplemental methods provides additional details on all methods described above.

## Supplementary Information

Supplementary Information.

## Data Availability

The datasets generated and analyzed during the current study are not publicly available due to confidentiality and some having been originally generated by the researchers of the ZamPHIA project but are available from the corresponding author on reasonable request.
