# Supplementary material for: Leveraging a national biorepository in Zambia to assess measles and rubella immunity gaps across age and space
Source: Sci Rep. 2022 Jun 17;12:10217. doi: 10.1038/s41598-022-14493-3 (PMC9204687; doi:10.1038/s41598-022-14493-3)
Supplement: Supplementary file 1 — Supplementary Information. [file 41598_2022_14493_MOESM1_ESM.pdf]

Supplemental Methods

Leveraging a national biorepository in Zambia to assess measles and rubella immunity gaps across age and space

Andrea C. Carcelen[a*], Amy K. Winter[b*], William J. Moss[a,c,d] Innocent Chilumba[e], Irene Mutale[e], Gershom Chongwe[e], Mwaka Monze[f], Gina Mulundu[f], Hope Nkamba[f], Francis. D. Mwansa[g], Lloyd Mulenga[h], Dale A. Rhoda[i], Kyla Hayford[a^], Simon Mutembo[a,g^]

* co-first authors, equal contribution
^ co-senior authors, equal contribution

Subsample selection

We selected 11,500 specimens from the 25,383 specimens on the ZAMPHIA biorepository list, upon accessing the biorepository, it was noted that these were not all available. Table SM1 notes the specimens that no longer had specimen available in the biorepository, those that were not found, and those that prohibited further testing beyond the original intent. Specimens were selected based on HIV infection status, geographic cluster, and age. To ensure representation, all HIV-infected participants and participants from small clusters (≤10 participants) were selected for serologic testing. The remaining participants selected in a province were HIV-uninfected and selected from all other clusters. The proportion of HIV-infected individuals in the sample was trying to match that found in the biorepository. The 0-14 year old children sampled had 1% HIV infected, matching the 1% in the biorepository. Amongst adults 15-49, 7% of our sample had HIV, compared to 12% in the biorepository. This was due in part to having no remaining specimen for testing.

To ensure sufficient sample size for age-specific seroprevalence estimates, participants younger than 5, 5-9, and 10-14 years of age were selected to each represent 22% of the subsample in each province, and participants 15-19 and 20-49 years were selected to each represent 17% of the subsample in each province (Fig. SM1). At least one respondent from each cluster was included. Not all selected specimens were able to be tested due to non-availability (14%) or lack of consent for future testing (<1%).

Status	N	%
Testing Complete	9,854	85.7
No available specimen	1,623	14.1
No consent for additional testing	6	0.1
Specimen not found	17	0.1

Table SM1. Availability of 11,500 samples selected for testing

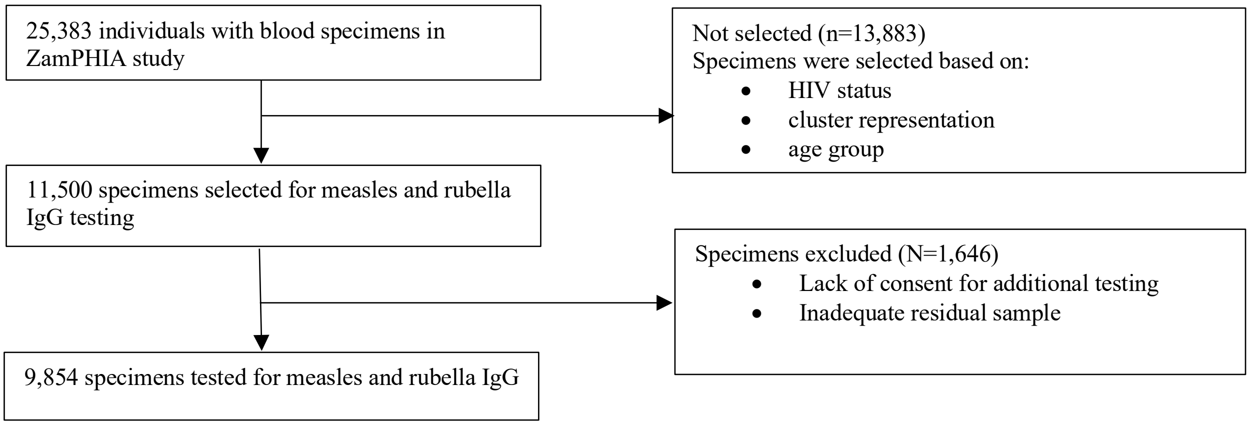

Figure SM1. Flow diagram for the selection of specimens from biorepository into subsample of specimens that were tested for measles and rubella antibodies.

Weighting

We calculated a weight for each specimen to be used in survey estimation where the confidence intervals were calculated using linearized Taylor series variance estimation. The weight was calculated based on probability of selection, nonresponse, trimming, and post-stratification.

Probability of selection

Weights from the ZAMPHIA project for all 11,500 PIRMZ samples selected were post-stratified to be nationally representative by scaling them to match 2016 population projected estimates from the Zambia Central Statistical Office in each stratum defined by province and age group.[1] These representative weights were inverted to calculate the cumulative probabilities of selection for each stage of the ZAMPHIA project. Those cumulative probabilities were multiplied by the probability of PIRMZ selection from the ZAMPHIA dataset. The updated product represents the cumulative probability of selection of all stages of ZAMPHIA and selection for PIRMZ. The updated cumulative probabilities were inverted once again to construct PIRMZ base weights.

Nonresponse

We adjusted for nonavailability of specimens through a nonresponse adjustment. To maintain the representativeness of the PIRMZ sample, the sampling weights were adjusted to account for blood non-availability and for non-consent for further testing. Logistic regression indicated that availability of specimens differed to a statistically significant degree across adjustment cells defined by province and age group but not by sex. Adjustment cells were defined by province and age group, and within each cell, the sum of weights from all non-respondents (those without specimens available for testing) were shifted to respondents with specimens available. The weights of available specimens were adjusted upward and the weights of non-available specimens were set to zero. After this adjustment, the sum of weights in each adjustment cell was equal to the sum of weights before the adjustment, but after the adjustment, all the weight is carried by available specimens that were successfully tested in the PIRMZ study.

Trimming weights

To be consistent with the weighting procedures from ZAMPHIA, weights that were larger than 3.5 times the median weight value in strata defined by province, age group, and sex were trimmed back and set equal to 3.5 times the median. A total of 47 respondents had outlier weights that were trimmed; all other weights remained the same.

Post-stratification

Finally, the trimmed weights were post-stratified so the sum of weights for specimens with PIRMZ testing results in each stratum defined by province, sex, and age group would equal the population age structure projections for 2016 provided by Zambia's Central Statistical Office, which were based on the 2010 census.[1]

Laboratory methods

Description of laboratory procedures

Specimens were tested using a boxwise testing strategy, where one freezer box of specimens was tested on each ELISA plate. Measles and rubella IgG ELISA testing was conducted simultaneously by different technicians according to manufacturer's recommendations. The optical density (OD) was read at 450 nm and 620 nm using a BioTek ELx800 microplate reader (BioTek Instruments, Winooski, VT). Plate-specific standard curves were used to calculate quantitative antibody concentrations from the OD values, per the manufacturer's recommendations. Qualitative designations were based on these values.

Enzyme immunoassays (EIAs) for antimeasles IgG (catalog no. EI2610-9601G; Euroimmun, Lubeck, Germany) and antirubella IgG (catalog no. EI2590-9601G; Euroimmun, Lubeck, Germany) were used per manufacturer's instructions for all experiments. Reported sensitivity and specificity are 100% for both for measles, but the measles kit has not been included in other reviews of EIA kits.[2] The rubella kit reports 99.6% sensitivity and 100% specificity. The rubella kit has also been compared with the Enzygnost ELISA, and showed very high agreement and 95.9% sensitivity and 100% specificity for IgG detection.[3]

The original qualitative interpretation for the measles IgG EIA kits were revised by the manufacturer for population-level seroprevalence studies like ours. Whereas the original values of ≥275 mIU/ml were classified positive, values of ≥200 to <275 were classified equivocal, and values of <200 were classified negative; we used the revised thresholds of ≥200 mIU/ml were classified positive, values of ≥150 to <200 were classified equivocal, and values of <150 were classified negative

Measles and rubella samples above the top calibrator 1 were set at the upper limit of detection (5,000 mIU/ml and 200 IU/ml respectively). Similarly, samples below the lower limit of detection were set to the lower limit of detection (8 mIU/ml and 0.3 IU/ml respectively).

Quality control and quality assurance

We monitored laboratory results in near time, analyzing uploaded data daily. This included checking for plate validity, intra-plate concordance and inter-plate concordance. We also monitored inter-technician variability and inter-laboratory variability. The Euroimmune IgG ELISA kit contains positive and negative controls that serve as internal controls for the reliability of test procedures. We added 2 additional internal controls (one high positive and one equivocal/negative for measles and rubella) to every plate to monitor reliability across all the plates. Invalid plates, as defined by the manufacturer were rerun.

We systematically retested specimens to assess intra- and inter-plate variability and reproducibility. Eight specimens on each plate were predefined to be run in duplicate for intraplate variability. Mean coefficient of variation for measles IgG mIU/mL intraplate retesting was 0.07 (SD=0.09) [median=0.05, IQR: 0.02-0.09] with a correlation coefficient (R) of 98.7% and for rubella was 0.00 (SD=2.83) [median=0.14, IQR: 0.06-0.30] with R of 99.0%. Four specimens from each plate were predefined to be rerun on a separate plate for retesting. Mean coefficient of variation for measles IgG interplate retesting was 0.10 (SD=0.12) [median=0.06, IQR: 0.03-0.13] with R of 91.3% and for rubella was 0.09 (SD=1.23) [median=0.06, IQR:0.02-0.15] with R of 97.1%. Coefficient of variation <10 was considered good, 10-14 acceptable, and ≥15 unacceptable.

Equivocal results were re-tested and, classified based on the positive or negative value on retesting. If equivocal on retest, they remained classified as such. Equivocal results were categorized as positive for

binary analyses. If there was discordance between positive and negative results upon retest, specimens were retested a third time and classified based on the two concurrent results.

Performance of DBS compared to plasma

Because only DBS were available for participants under 2 years of age, we validated that the DBS provided consistent results with plasma. We selected 100 plasma specimens that had paired DBS available, because they were prepared as part of HIV testing procedures for ZamPHIA, and tested both the plasma and DBS to compare.

We tested 3 different protocols to optimize the protocol for DBS elution.[4] The final protocol involved the circumference of each DBS being measured, punched with sterilized 6mm hole punch, and serum being eluted with 450 μL of buffer, adapted from the manufacturer's recommendation.[5] One hundred microliters of eluted sample were transferred to precoated 96-well plates and the manufacturer's protocol was followed to perform the EIA, as was done with plasma.

The results demonstrated consistent readings across both the DBS and plasma for measles and rubella IgG (Fig. SM2). For measles, R2=97.2% and mean coefficient of variation is 0.09 (SD=0.09) [median=0.06, IQR: 0.04-0.13]. For rubella, R2=98.3% and mean coefficient of variation is -0.27 (SD=2.06) [median=0.04, IQR:-0.12-0.11].

These results suggested no adjustments were needed for the DBS tested. There were similar findings in India, where sensitivity of antibody detection by DBS was greater than 98%, and specificity was 90% and 98%, for measles and rubella IgG, respectively.[6]

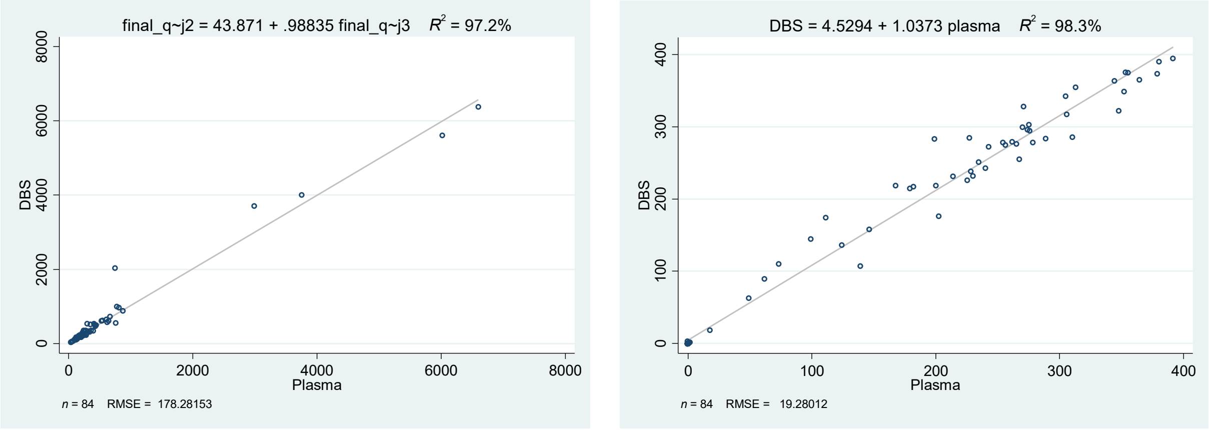

Figure SM2. Measles (left) and rubella (right) comparisons between DBS and plasma ELISA IgG results. The regression line for measles is represented by y=43.87+0.99x. The regression line for rubella is represented by y=4.53+1.04x.

<u>Estimating Measles and Rubella National & Provincial Age-Specific Seroprevalence</u>

The nested serosurvey was sampled to represent provinces by age-group, therefore to characterize age-specific curves by province we took into account the hierarchical structure of the data. We fit hierarchical generalized additive models to individual measles and rubella seropositivity by age. The evaluated models accounted for the data structure by including a single national level smoother over age applied to all provinces.[7]

$$g(\Pr(seropos_i)) = f(age_i) + f_{province_i}(age_i)$$

where g is a link function, f is a smoothing function over age, and $f_{province}$ is a smoother for age for a given province. Specimens from individuals younger than 9 months of age with potential maternally-derived antibodies were excluded to improve model fit. The R package *mgcv* was used to fit the hierarchical generalized additive models given its computational efficiency, automated selection of the smoothness parameter, and goodness of fit to the data.[8] The models assumed a binomial probability distribution for seropositivity and included isotropic smoothers on age. We compared models with different smoothing bases (cubic regression splines and thin plate regression splines), and with different link functions (complementary log-log and log odds). The choice of the basis dimension in each model was large enough to have sufficient degrees of freedom to represent the underlying data.[8] Final models for measles and rubella were selected by minimizing the Akaike Information Criterion (AIC).

Using Indirect Methods to Reconstruct Measles National Age-Specific Immunity Profiles

In addition to the directly estimated national age-specific profile of measles immunity using serological data described above, we also indirectly reconstructed national age-profile of measles immunity. The indirect analysis, modified methods from Takahashi et al. 2015,[9] estimates the proportion of each birth cohort that is immune based on probabilities of vaccination (routine and campaigns) taking into account vaccine effectiveness, risk of natural infection, and probability of maternally derived immunity. For example, if 80% of the birth cohort was routinely vaccinated and the cumulative measles attack rate was 75% among those unvaccinated, then 95% of the cohort would be estimated as immune and 5% would be susceptible. This is illustrative, and a simplification as it assumes 100% vaccine effectiveness by age, a time constant cumulative force of infection, and no opportunity for a vaccination campaign.

We assumed that routine vaccination coverage rates were equivalent to the World Health Organization and United National Children's Fund (WUENIC) estimates for routine MCV1 and MCV2 coverage rates for Zambia 1983-2016 and 2014-2016, respectively.[10] We assumed vaccine effectiveness for MCV1 and MCV2 was 85% and 95%, respectively.[11] We assumed dependence between routine doses such that the probability of being successfully routinely vaccinated within a birth cohort was (MCV1.VE*MCV1.Coverage) + (MCV1.Coverage*(1-MCV1.VE)*MCV2.Coverage* MCV2.VE), where VE stands for vaccine effectiveness.

Vaccination campaign timing, age ranges, and coverage rates were extracted from WHO reported administrative estimates.[12] Zambia has conducted four national measles vaccination campaigns (Table SM2), we assumed maximum coverage of a campaign was 99%. We assumed campaign vaccine effectiveness of 95% among birth cohorts who were at least 12 months at the time of the campaign. If the campaign minimum target age was 6 or 9 months old we assumed 74% and 89% vaccine effectiveness among birth cohorts who were 6-11 or 9-11 months at the time of the campaign, respectively.[11] We assumed that if a birth cohort had experienced vaccination via a campaign as well as routine vaccination, the probability of successful vaccination in that cohort was taken as the higher of the two values: the probability of being successfully routinely vaccinated or the probability of being successfully vaccinated in a campaign. This simplification is similar to assuming 100% correlation between routine and campaign vaccination, however it does not allow for the probability of successful vaccination following a primary vaccine failure. Given the very high rates of campaign coverage, the results are robust to this analytic simplification.

Year	Region	WHO Reported Administrative Coverage	Assumed National Coverage	Targeted Age Range
2002	1 province	112%	-	6mo - 15yo
2003	9 provinces	108%	99%	6mo - 15yo
2007	national	107%	99%	9mo - 4yo
2010	national	115%	99%	9mo - 4yo
2012	national	116%	99%	6mo - 14yo

Table SM2. Zambia Measles Vaccination Campaigns prior to 2016 per [12]

The proportion of infants with maternally derived immunity was estimated as the mean of exp $(-0.45a)$, where a is age in months from 1 to 12.[13] This is equal to 14.6% of the infant population with maternally derived immunity.

The probability of immunity from natural infection for each birth cohort was estimated using the catalytic model taking into account the annual forces of infection each birth cohort was exposed to over their lifetime. The probability of immunity for each age *a* is defined as,

$$Pr(infection\ by\ age\ a) = 1 - e^{-\sum_{t=(2016-a)}^{t=2016} \lambda_t}$$

where λ_t is the annual force of infection at year *t*. We estimated Zambia's year-specific force of infection from 1981 to 2016 using Simons et al 2012 state space model fit to reported measles cases.[14] This model corrects for under-reporting of measles cases and estimates annual measles cases, in addition to annual number of susceptible individuals and infectiousness. The year specific force of infection (λ_t) is calculated from the state space model outputs defined as $\lambda_t = \theta_1 \times S_t/N_t$, where S_t is time-specific number of susceptible individuals, N_t is the total population over time, and θ_1 is an estimated infectiousness parameter. We assumed an annual force of infection of 0.3 for all years prior to 1981, equivalent to about 90% of infections occurring prior to 10 years of age.

Using Indirect Methods to Reconstruct Provincial Measles Immunity Profiles in Individuals 10 months to 4 years old

To reconstruct provincial age-specific immunity profiles we estimated the proportion of each birth cohort that is immune based on routine vaccination only, taking into account vaccine effectiveness. We focused on birth cohorts born 2013 to 2016 who would not have been eligible for the 2012 vaccination campaign and assumed they had no risk of natural infection over this time period given the small number of measles cases reported in Zambia since 2016 (an average of 11 annual reported cases between 2016 and 2019).[15] We relied on Zambian administrative measles vaccination data (i.e., the reported number of measles vaccines for each dose delivered each year) by province as the numerator by which to estimate vaccination coverage per birth cohort. We relied on population projections using a cohort component model calculated by the Zambian Statistical Office for total and age-specific provincial population sizes to estimate the denominator (i.e., the number eligible for each vaccine dose).[1] The "traditional" method used by the Zambian EPI program, assumes that the population eligible for MCV1 vaccination is 4% of the total provincial population size assuming a homogeneous birth rate of close to 40/1000 population and ignoring mortality before the age for routine MCV1, and the population eligible for MCV2 vaccination is 8% of the total provincial population size. The "revised" method assumes that the population eligible for MCV1 vaccination is the number of individuals listed as age 0 in each province (which takes into account province-specific births and infant mortality rates), and the population eligible for MCV2 vaccination is the number of individuals listed as age 1 in each province. We assumed routine doses were dependent (treating MCV2 as a true second dose) and that vaccine effectiveness for MCV1 and MCV2 was 85% and

95%, respectively.[11] We are estimating immunity for individuals over 9 months old, therefore do not consider maternally derived immunity.

Measles District Age-Specific Seroprevalence

To estimate district-specific measles seroprevalence, hierarchical spatial models were fit to individual measles seropositivity. District-specific random effects were included in the model based on a conditional autoregressive (CAR) specification in which districts adjacent to one another were assumed to be more similar than districts not adjacent. The models assumed a binomial probability distribution for seropositivity and a log odds link.

We explored epidemiological and demographic model covariates. Covariates of HIV positivity, age, district, and province of residence were extracted from the ZAMPHIA questionnaire and linked directly to the serum samples. We explored demographic and measles epidemiological covariates that were linked to serum samples based on the age and district of residence of the individual sampled. These included covariates from Zambia's EPI program such as district and year-specific MCV1 and MCV2 administrative coverage or eligibility, district and year-specific outbreak risk defined as at least one, two, or three annual measles-specific IgM positive cases, and campaign coverage eligibility and province-specific 2012 vaccination campaign coverage (we did not have access to province-specific campaign coverage for campaigns prior to 2012). We also evaluated district specific population density, district and age-specific MCV1 vaccination coverage from Zambia's 2018 Demographic and Health Survey (DHS) that we estimated using geospatial modeling techniques,[9] and district-specific MCV1 vaccination coverage estimates per Institute for Health Metrics and Evaluation Global Health Data Exchange.[16] The final model, described below, was selected by minimizing the Widely Applicable Information Criterion (WAIC).

$$\begin{aligned} logit(Pr(seropos_{ijk})) = \gamma_j + \beta_0 HIVpos_i + \beta_1 HIVpos_i * age_i + \beta_2(1 - HIVpos_i) * age_i + \\ \beta_3 HIVpos_i * age_i^2 + \beta_4(1 - HIVpos_i) * age_i^2 + \\ \beta_5 under4_i * mcv1_i + \beta_6 mcv2eligible_i * mcv2_i + \\ \beta_{7,k} SIAeligible_i + \beta_8 Outbreak_i \end{aligned}$$

Indexes *i, j,* and *k* represent the individual, district, and province from which the serum sample was collected. *HIVpos* is a binary variable based on HIV positivity, *age* is age in years, *mcv1* is the MCV1 vaccination coverage estimated from DHS data, *under4* is a binary variable classifying individuals under four years of age, *mcv2eligible* is a binary variable indicating whether the individual was eligible for MCV2 (introduced in 2012), *mcv2* is MCV2 vaccination coverage estimated from administrative data, *SIAeligible* is a binary variable classifying an individual as eligible for the 2012 measles vaccination campaign that targeted individuals 6 months through 14 years old, *Outbreak* is a binary variable indicating whether an individual was exposed to a district-specific measles outbreak since 2012. We allowed provincial specific effects of the 2012 vaccination campaign eligibility. We defined an outbreak within a district as two or more measles-specific IgM positive cases reported within a year based on data collected by Zambia EPI program. We also included interactions between HIV serostatus and age, as well as HIV serostatus and squared age.

To estimate γ_j we assumed that it is multivariate normally distributed with a location-specific mean of $\mu_{x,i} = 0$ and a spatially-independent standard deviation of conditional autoregressive model (CAR) specification for the spatial random effects, parametrized by the precision matrix, $1/\sigma_x^2$:

$$\gamma_j \sim Normal(\mu_{x,i}, \sigma_x^2)$$

$$1/\sigma_x^2 = \tau_x(D - \alpha_x W)$$

Above, we have the precision matrix $1/\sigma_x^2$, τ_x is a precision parameter, α_x controls the spatial dependence (α_x=0 implies spatial independence, and α_x=1 collapses to an intrinsic conditional autoregressive model), D is an j by j diagonal matrix with diagonal elements encoding the number of adjacent neighbors that each district has, W is a binary adjacency matrix. This specification means that estimated seroprevalence at any given district is conditional on the estimated seroprevalence of neighboring districts.

The model was fit using Stan in R using two chains with 5000 iterations per chain (half of all iterations were burn-in) until model convergence was achieved.[17] The posterior distribution of alpha collapsed to 1, resulting in an intrinsic conditional autoregressive model (Fig. SM3). Figure SM4 displays model estimates of gamma (district-level intercepts), where we find intra and inter-district variation. The partial pooling or regularization effect of the hierarchical spatial model is displayed in Figure SM5; districts with low seroprevalence are pulled upwards and districts with high seroprevalence are pulled downward, shrinking all estimates towards the mean. Figure S6 displays estimates for the beta parameters. HIV positive individuals had significantly lower seroprevalence (beta0), and among individuals less than four years old we find individuals exposed to higher vaccination coverage rates within the respective district and birth cohort had significantly higher seroprevalence (beta5). The impact of being eligible for the most recent SIA on seroprevalence differed by province (beta7); Northern and Western provinces had a larger positive impact of the SIA than other provinces in the country.

We conducted leave-one-out cross validation analyses to evaluate the performance of the model to estimate district and age-specific seroprevalence. In these analyses we left out each district or age in years, retrained the model with the smaller dataset, and then used the new posterior estimates of the parameter values to predict seroprevalence for the district or age originally left out (Fig. SM7-SM8). The model does well to predict seroprevalence for missing ages, but not to predict seroprevalence for missing districts. This finding is expected, given our reliance on the model district-specific random effects (gamma parameter) that captures variation not explained by our demographic and epidemiologic covariates. We find that extrapolating the gamma parameter for a missing district does a poor job to capture observed district seroprevalence. Fortunately, we do not extrapolate our model to predict seroprevalence any new districts, and only extrapolate to missing ages within a district. As a result, the model is suitable to answer the question at hand (i.e., district and age-specific seroprevalence for the 72 districts represented in the data).

The final step to estimate district-specific seroprevalence is to weight the seroprevalence estimates by district-specific population characteristics. We created a new dataset with all possible covariate groupings. For example, one possible covariate grouping is district Chadiza, HIV negative, 7 years old which is associated with not being under four years old, not eligible for MCV2, eligible for the 2012 SIA in Eastern Province, and exposure to a local outbreak. We estimated the probability of seropositivity for each covariate grouping across 2500 samples from parameter posterior distribution sets, taking into account both uncertainty of the mean parameter values and uncertainty in the sampling process. We then weighted the probability of seropositivity by each covariate grouping and sampled parameter set per district by the proportion of individuals in that covariate grouping to get 2500 estimates of seroprevalence for each district. Figure 1C in the main text shows the final results.

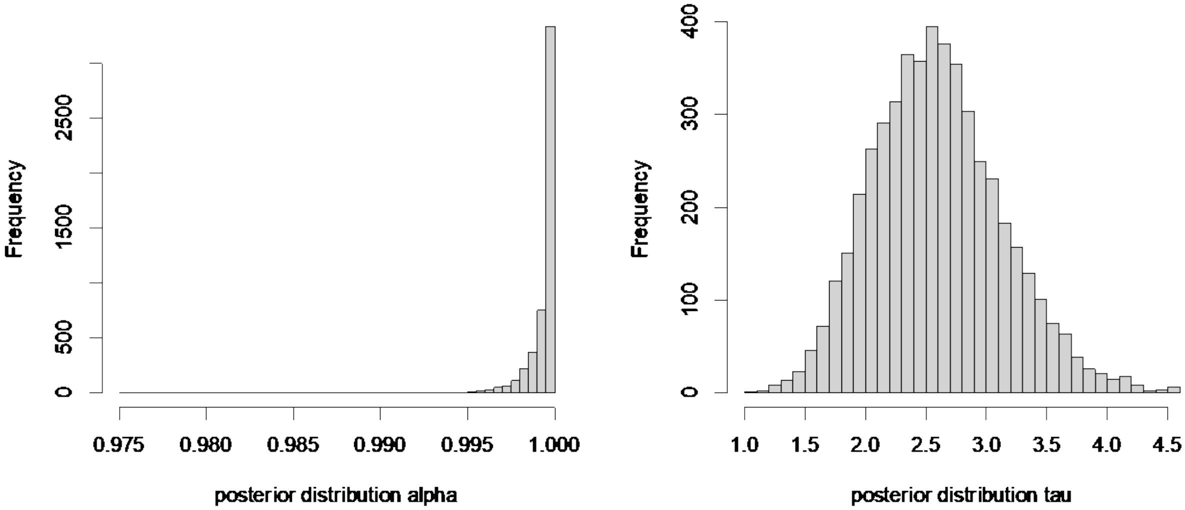

Figure SM3. Posterior distribution of alpha and tau parameters.

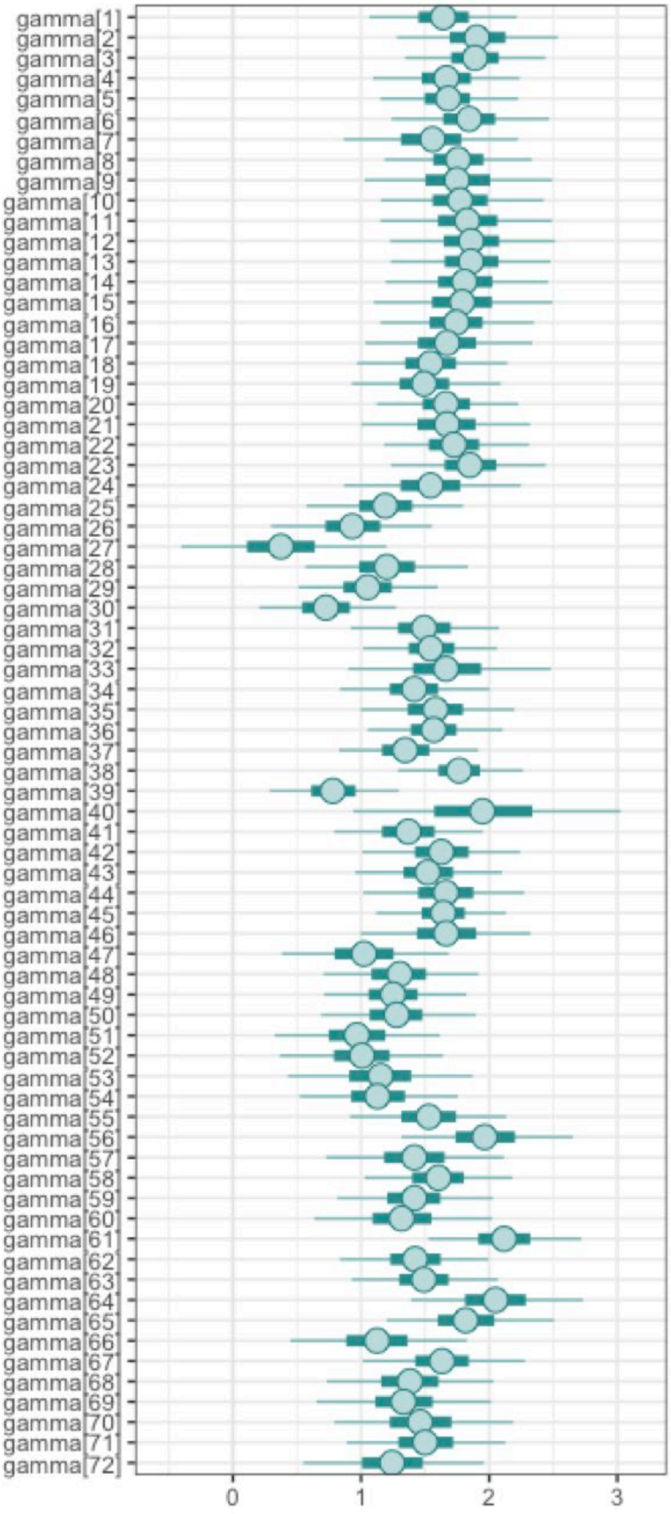

Figure SM4. Final model output. Parameter estimates mean (point), 50% (thick line) and 95% (thin line) credible intervals for gamma parameters (district-level intercepts).

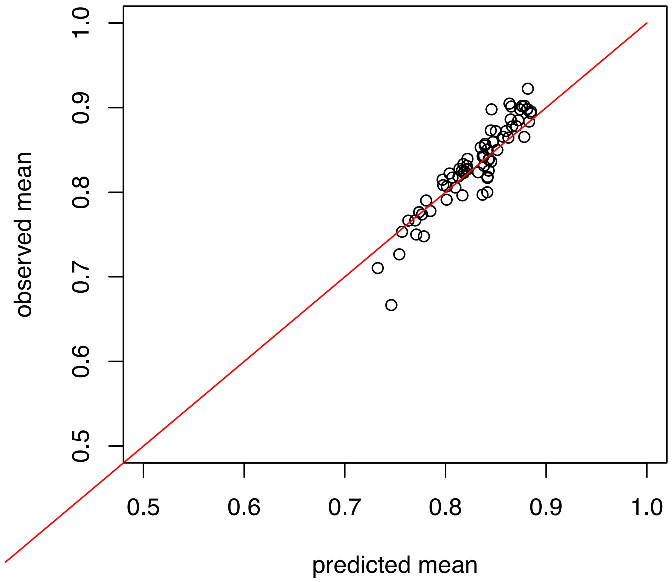

Figure SM5. Observed vs predicted district specific measles seroprevalence. Each point represents a different district. The red solid line represents perfect agreement.

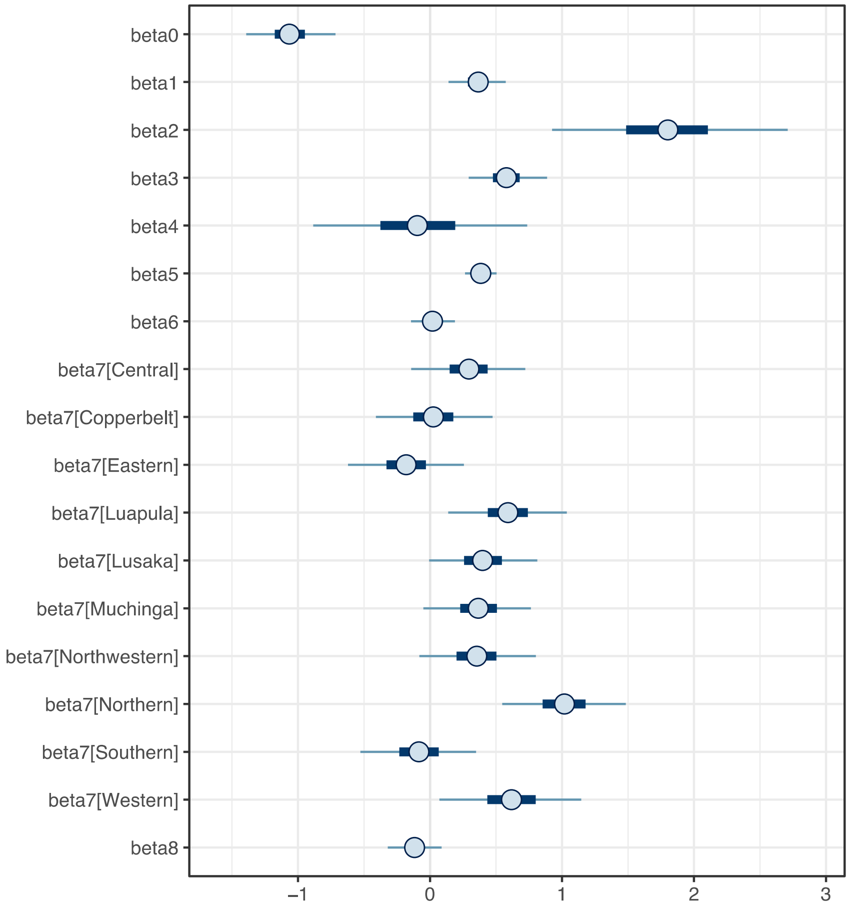

Figure SM6. Final model output. Parameter estimates mean (point), 50% (thick line) and 95% (thin line) credible intervals for beta parameters.

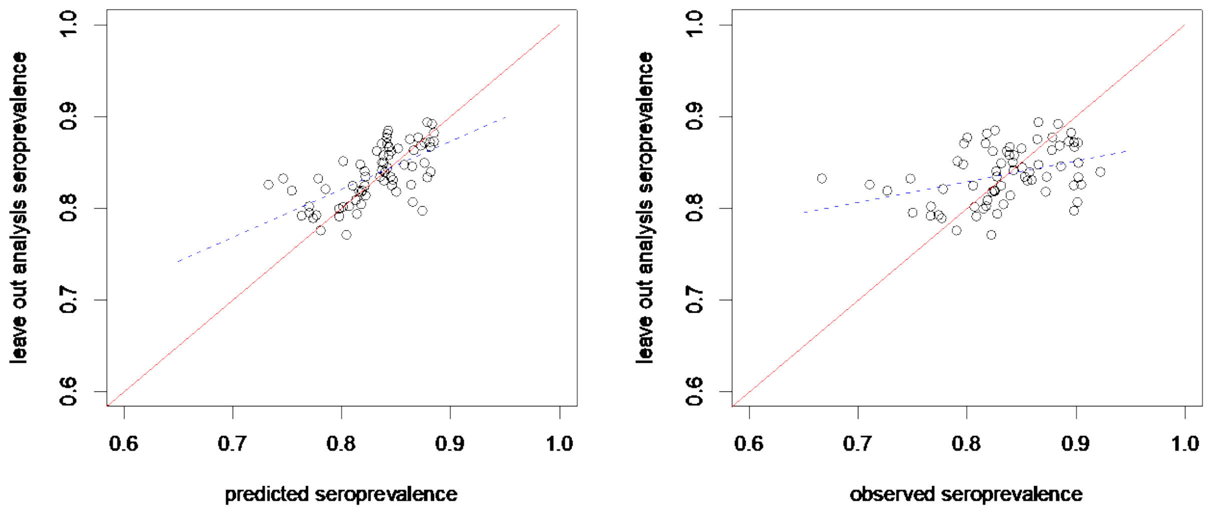

Figure SM7. Results of leave out district analysis. Each point represents a different district. Left figure displays the estimated mean seroprevalence for a predicted districts left out of the analysis by the

expected seroprevalence given the district was included in the analysis. Right figure displays the estimated mean seroprevalence for a predicted districts left out of the analysis by the observed seroprevalence for the respective district. Dashed blue line is fit line and red solid line represents perfect agreement.

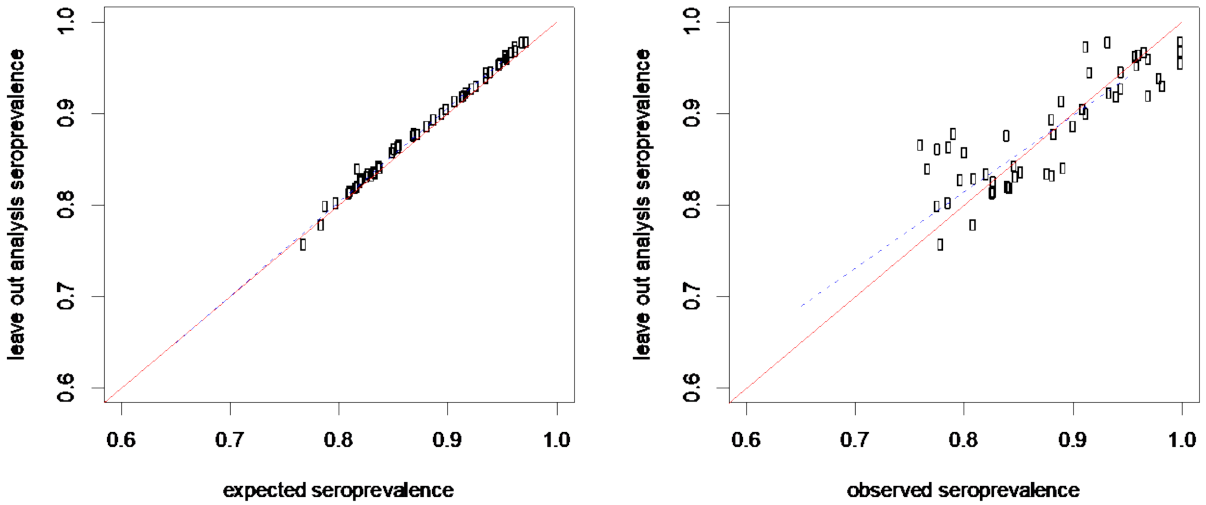

Figure SM8. Results of leave out age analysis. Each point represents a different age in years. Left figure displays the estimated mean seroprevalence for a predicted ages left out of the analysis (y-axis) by the expected seroprevalence given the age was included in the analysis (x-axis). Right figure displays the estimated mean seroprevalence for a predicted ages left out of the analysis (y-axis) by the observed seroprevalence for the respective age (x-axis). Dashed blue line is fit line and red solid line represents perfect agreement.

Measles Outbreak Risk 2016-2019

We evaluated Zambia's national measles outbreak risk 2016 to 2019 by estimating measles effective reproduction number (R_{eff}) 2016 to 2019. R_{eff} is the average number of secondary cases per infectious individual. If R_{eff} is over one, cases can increase and there is risk of an outbreak. If R_{eff} is less than one, the number of cases will decline and transmission will eventually cease. We estimate R_{eff} as the dominant eigen value of the next generation matrix, **K**.[18] The next generation matrix is defined as

$$\mathbf{K} = \mathbf{s} \times (1 - e^{-\mathbf{W}/\mathrm{N}})$$

where $\mathbf{s} = (\mathrm{s}_1, \mathrm{s}_2, \mathrm{s}_3, \ldots \mathrm{s}_\mathrm{n})$ as a vector of number susceptible individuals in *n* age groups, N is the total size of the Zambian population under 50 years old, and **W** is the who acquires infection from whom (WAIFW) matrix that is *n* x *n* dimensions. The WAIFW matrix was calculated by scaling an inferred age-contact matrix for Zambia derived by Prem et al. 2017 [19], such that the dominant eigen value of the next generation matrix calculated from the inferred age-contacts and 2015 Zambian age structure was equal to the conservatively assumed measles basic reproduction number of 12 (although estimates of the basic reproduction number vary substantially [20]). We assumed **W** was constant between 2016 and 2019; however we estimated N and **s** each year 2016 to 2019. The total size of Zambia's population under 50 years old from 2016 to 2019 was estimated from United Nations Population Projections for Zambia.[21] The number of susceptible individuals per age group in 2016 was estimated directly from the 2016

seroprevalence data and Zambia's estimated age structure per United Nations Population Projections.[21] The number of susceptible individuals per age group in 2017 to 2019 was indirectly estimated from the 2016 seroprevalence data.

Methods developed by Funk et al. 2019 relied on cross-sectional serological data to estimate future seroprevalence.[22] This innovative approach extends the utility of seroprevalence data beyond the year of collection, and was relied on to estimate measles national age-specific seroprevalence 2017 to 2019. We focused on immunity due to vaccination only, assuming immunity due to natural infection would have no meaningful impact at the population level given the low number of measles cases reported in Zambia since 2016 (an average of 11 annual reported cases between 2016 and 2019).[15] We assumed that immunity for new birth cohorts eligible for routine MCV1 was given by a routine vaccination scaling factor multiplied with the reported coverage in that year. We assumed that additional immunity from the 2016 campaign occurring after serological data collection was given by a vaccination campaign scaling factor multiplied with the reported campaign coverage. The scaling factors were estimated as the ratio of the observed seroprevalence for eligible birth cohort(s) and the level of reported coverage. For the 2016 campaign and second dose of measles, we assumed that the vaccine was preferentially given to those that had received the first dose of the vaccine. For the routine measles second dose we additionally took into account vaccine effectiveness. MCV1 and MCV2 coverage rates for Zambia were extracted from the World Health Organization and United National Children's Fund (WUENIC) estimates.[10] The 2012 and 2016 measles vaccination campaign coverage estimates were extracted from WHO survey estimates of 96% and 95%, respectively.[12] By relying on scaling factors for routine MCV1 and campaign, we are assuming i) the relationship between estimated coverage and seroprevalence remains constant over time and ii) that seropositivity is only a result of the routine or campaign vaccination. The result of assumption ii is likely to under-estimate the scaling factor and over-estimate the impact of vaccinations on seroprevalence; therefore reducing estimates of R_{eff}. Additionally, the conservative assumption of scaling the WAIFW to a basic reproduction number of 12 rather than 15 or 20 or higher, also results in a lower estimated $R_{eff.}$

Rubella Basic Reproductive Number and Congenital Rubella Syndrome Incidence Rate

Rubella basic reproductive number was estimated at the national level, defined as G/A, where G is the reciprocal of Zambia's 2016 per capita birth rate (38.41 per 1000) and A is the average age of rubella infection. The average age of infection, A can be directly estimated from age-specific seroprevalence data by taking the integral of the proportion susceptible $A = \int_{0.83}^{49}(1 - \pi(a)da,$ where $\pi(a)$ is age-profile of proportion seropositive estimated from the generalized additive model above, a is age in years and ranging from 0.83 (i.e., 10 months old to ignore passively acquired immunity) to age 49. We estimated the average age was infection of 8.59 (95% CI 6.5, 10.77).

The estimated CRS rate (CRS incident cases per 100,000 live births) for each reproductive age in years (15-49) and province was estimated by

$$CRSrate_{a,p} = (1 - \pi_{a,p}) \times (1 - exp^{-16\lambda_{a,p}/52}) \times 0.65 \times 100{,}000,$$

where $\pi_{a,p}$ is the estimated seroprevalence at age a and province p, $\lambda_{a,p}$ is the estimated force of infection at age a and province p. The force of infection was defined as $\pi_{a,p}'/\ (1 - \pi_{a,p})$.[23] We assumed that 65% of infants born to women infected during the first 16 weeks of pregnancy would be born with CRS.[24]

The provincial total CRS incidence per 100,000 live births was calculated as the mean of CRS rate in each reproductive age in years weighted by the number of births in each reproductive age in years. It is defined as

$$CRSrate_p = \sum_{a=15}^{a=45} (CRSrate_{a,p} \times b_{a,p}/\sum_{a=15}^{a=45} b_{a,p}),$$

where $b_{a,p}$ is the annual number of births to women of age a and province p. The annual age and province specific number of births was estimated using Zambia's Central Statistical Office projected 2016 provincial total number of births and national age-specific fertility rates,[1] such that the mothers' age distribution of the total provincial number of births was determined by the national age specific fertility rate.

Chronology and operationalization of serosurvey

Using a pre-existing biorepository had its own challenges even if the complexities of specimen collection were circumvented. Additional policies and procedures required to access specimens collected by another organization had to be fulfilled. These included making amendments to the original protocol and obtaining ethical approvals from all the institutions that were involved in the original ZAMPHIA study. By the time of our study the procedures for accessing the biorepository were not yet in place and this added an extra layer of complication and required a lot of negotiating with all the ZAMPHIA stakeholders. Additionally, interpreting and accessing sociodemographic data from the survey was delayed due to unclear policies and coordination between organizations. The table below summarizes timing of study activities (Table SM3). These HIV Impact Assessments are being conducted in at least 15 low and middle income countries, so this process could be replicated.[25]

**Study activity**	**Approximate timeline**
Ethical approvals	November 2017-June 2018
Accessing and subsampling specimens	August-October 2018
Laboratory training and specimen organization	November 2018-March 2019
Laboratory Testing	April-October 2019
Accessing and linking sociodemographic data	July-November 2019
Data analysis and modeling	October 2019-April 2020
Report writing and presentation development	December 2019 (preliminary presentation)-May 2020

Table SM3. Timeline of activities

References

1. Zambia Statistics Agency. *Zambia Population and Demographic Projections, 2011-2035.* https://www.zamstats.gov.zm/index.php/publications/category/3-census-statistics (2020).
2. Latner, D. R. *et al.* Qualitative Variation among Commercial Immunoassays for Detection of Measles-Specific IgG. *J Clin Microbiol* **58**, e00265-20 (2020).
3. Viswanathan, R. *et al.* Comparison of two commercial ELISA kits for detection of rubella specific IgM in suspected congenital rubella syndrome cases and rubella IgG antibodies in a serosurvey of pregnant women. *Diagn Microbiol Infect Dis* **94**, 243–247 (2019).

4. Kaduskar, O. *et al.* Optimization and Stability Testing of Four Commercially Available Dried Blood Spot Devices for Estimating Measles and Rubella IgG Antibodies. *mSphere* e0049021 (2021) doi:10.1128/mSphere.00490-21.
5. World Health Organization. *Annex A4 Dried blood spot (DBS) protocols*. https://www.who.int/immunization/monitoring_surveillance/burden/laboratory/AnnexA4_DBS_protocols_v1_7_Jan_2020_jr.pdf?ua=1 (2020).
6. Prosperi, C. *et al.* Diagnostic Accuracy of Dried Blood Spots Collected on HemaSpot HF Devices Compared to Venous Blood Specimens To Estimate Measles and Rubella Seroprevalence. *mSphere* e0133020 (2021) doi:10.1128/mSphere.01330-20.
7. Pedersen, E. J., Miller, D. L., Simpson, G. L. & Ross, N. Hierarchical generalized additive models in ecology: an introduction with mgcv. *PeerJ* **7**, e6876 (2019).
8. Wood, S. N. *Generalized Additive Models: An Introduction in R*. (Chapman and Hall/CRC, 2006).
9. Takahashi, S. *et al.* Reduced vaccination and the risk of measles and other childhood infections post-Ebola. *Science* **347**, 1240–2 (2015).
10. World Health Organization & UNICEF. WHO/UNICEF estimates of national immunization coverage, estimates for 1980 to 2019. https://apps.who.int/immunization_monitoring/globalsummary/timeseries/tswucoveragemcv1.html (2020).
11. Gans, H. A. *et al.* Deficiency of the humoral immune response to measles vaccine in infants immunized at age 6 months. *JAMA* **280**, 527–532 (1998).
12. World Health Organization. Data, statistics and graphics | 4.1 Summary of Measles-Rubella Supplementary Immunization Activities, 2000-2021. https://www.who.int/teams/regulation-prequalification/eul/immunization-vaccines-and-biologicals (2020).
13. Lessler, J., Moss, W. J., Lowther, S. A. & Cummings, D. A. T. Maintaining high rates of measles immunization in Africa. *Epidemiology and Infection* **139**, 1039–1049 (2011).

14. Simons, E. *et al.* Assessment of the 2010 global measles mortality reduction goal: results from a model of surveillance data. *Lancet* **379**, 2173–8 (2012).
15. World Health Organization. Immunization, Vaccines and Biologicals: Surveillance for Vaccine Preventable Diseases. https://apps.who.int/immunization_monitoring/globalsummary/timeseries/tsincidencemeasles.html (2020).
16. Institute for Health Metrics and Evaluation (IHME). Low- and Middle-Income Country MCV1 Coverage Geospatial Estimates 2000-2019. https://doi.org/10.6069/VFWJ-JF14 (2020).
17. Stan Development Team. *RStan: the R interface to Stan*. (2020).
18. Diekmann, O., Heesterbeek, J. A. & Metz, J. A. On the definition and the computation of the basic reproduction ratio R0 in models for infectious diseases in heterogeneous populations. *J. Math. Biol.* **28**, 365–82 (1990).
19. Prem, K., Cook, A. R. & Jit, M. Projecting social contact matrices in 152 countries using contact surveys and demographic data. *PLoS Comput. Biol.* **13**, e1005697 (2017).
20. Guerra, F. M. *et al.* The basic reproduction number (R0) of measles: a systematic review. *Lancet Infect. Dis.* **17**, e420–e428 (2017).
21. United Nations Population Division. *wpp2019 World Population Prospects 2019. R package version 1.1-1*. (2020).
22. Funk, S. *et al.* Combining serological and contact data to derive target immunity levels for achieving and maintaining measles elimination. *BMC Med* **17**, 180 (2019).
23. Hens, N. *et al. Modeling infectious disease parameters based on serological and social contact data : a modern statistical perspective*. (Springer, 2012).
24. Vynnycky, E. *et al.* Using seroprevalence and immunisation coverage data to estimate the global burden of congenital rubella syndrome, 1996-2010: A systematic review. *PLoS One* **11**, e0149160 (2016).

25. Columbia University,. PHIA Project: Population Based HIV Impact Assessment. *PHIA Project* https://phia.icap.columbia.edu/.

Supplemental Results

Leveraging a national biorepository in Zambia to assess measles and rubella immunity gaps across age and space

Andrea C. Carcelen[a*], Amy K. Winter[b*], William J. Moss[a,c,d] Innocent Chilumba[e], Irene Mutale[e], Gershom Chongwe[e], Mwaka Monze[f], Gina Mulundu[f], Hope Nkamba[f], Francis. D. Mwansa[g], Lloyd Mulenga[h], Dale A. Rhoda[i], Kyla Hayford[a^], Simon Mutembo[a,g^]

* co-first authors, equal contribution
^ co-senior authors, equal contribution

**Province**	**Measles seroprevalence** mean (95% CI)	**Measles significance**	**Rubella seroprevalence** mean (95% CI)	**Rubella significance**
Lusaka	82.9% (79.1%, 86.7%)		78.9% (75.9%, 82.1%)	reference
Central	84.7% (81.8%, 87.6%)		70.1% (66.7%, 73.5%)	***
Copperbelt	82.7% (79.2%, 86.1%)		76.3% (73.0%, 79.5%)	
Eastern	80.5% (77.4%, 83.7%)	*	74.9% (71.8%, 78.0%)	
Luapula	78.2% (75.2%, 81.4%)	**	74.4% (71.1%, 77.8%)	*
Muchinga	86.1% (83.8%, 88.3%)		75.4% (72.5%, 78.3%)	
Northern	87.0% (84.3%, 89.7%)	reference	75.6% (72.0%, 79.1%)	
North-Western	83.2% (80.2%, 86.2%)		72.5% (69.2%, 75.8%)	**
Southern	81.0% (78.0%, 84.0%)	*	72.2% (69.1%, 75.3%)	**
Western	83.4% (80.3%, 86.7%)		72.9% (69.0%, 76.8%)	*

Table S1: Measles and rubella survey weighted seroprevalence by province
significance levels: *** 0.001; ** 0.01; * 0.05

**Age group**	**Measles seroprevalence** mean (95% CI)	**Measles significance**	**Rubella seroprevalence** mean (95% CI)	**Rubella significance**
0-4	71.2% (68.9%, 73.6%)	reference	27.3% (25.0%, 29.6%)	reference
5-9	81.9% (80.1%, 83.7%)	***	65.1% (62.9%, 67.4%)	***
10-14	82.3% (80.5%, 84.0%)	***	82.3% (80.5%, 84.1%)	***
15-19	87.2% (85.5%, 88.9%)	***	91.2% (89.8%, 92.6%)	***
20-49	87.9% (85.4%, 90.3%)	***	95.7% (94.4%, 97.0%)	***

Table S2: Measles and rubella survey weighted seroprevalence by age group (years)
significance levels: *** 0.001; ** 0.01; * 0.05

**sex**	**Measles seroprevalence mean (95% CI)**	**Measles significance**	**Rubella seroprevalence mean (95% CI)**	**Rubella significance**
female	84% (82%, 86%)	reference	75% (74%, 77%)	reference
male	81% (80%, 83%)	*	74% (73%, 76%)	

Table S3: Measles and rubella survey weighted seroprevalence by sex. There is a significant interaction between sex and age (see Figure S2). significance levels: *** 0.001; ** 0.01; * 0.05

**Province**	**Age group**	**Measles seroprevalence** mean (95% CI)	**Rubella seroprevalence** mean (95% CI)
Lusaka	0-4	65% (58%, 72%)	27% (21%, 33%)
Lusaka	5-9	80% (75%, 85%)	67% (61%, 73%)
Lusaka	10-14	83% (79%, 88%)	86% (82%, 90%)
Lusaka	15-19	92% (88%, 95%)	94% (90%, 98%)
Lusaka	20-49	89% (81%, 96%)	98% (95%, 100%)
Central	<5	72% (65%, 80%)	27% (19%, 34%)
Central	5-9	86% (81%, 92%)	52% (44%, 60%)
Central	10-14	87% (82%, 92%)	74% (67%, 81%)
Central	15-19	89% (84%, 93%)	89% (85%, 93%)
Central	20-49	89% (83%, 94%)	95% (91%, 99%)
Copperbelt	0-4	74% (68%, 80%)	24% (19%, 30%)
Copperbelt	5-9	84% (80%, 89%)	70% (64%, 76%)
Copperbelt	10-14	82% (78%, 86%)	86% (82%, 90%)
Copperbelt	15-19	84% (78%, 90%)	93% (90%, 97%)
Copperbelt	20-49	86% (78%, 94%)	93% (87%, 99%)
Eastern	0-4	73% (66%, 81%)	29% (21%, 36%)
Eastern	5-9	81% (75%, 87%)	66% (59%, 73%)
Eastern	10-14	76% (70%, 81%)	80% (75%, 85%)
Eastern	15-19	79% (73%, 85%)	93% (89%, 96%)
Eastern	20-49	86% (80%, 93%)	97% (94%, 99%)
Luapula	0-4	58% (50%, 67%)	28% (21%, 36%)
Luapula	5-9	76% (69%, 84%)	68% (60%, 76%)
Luapula	10-14	81% (73%, 88%)	85% (79%, 91%)
Luapula	15-19	83% (78%, 89%)	91% (87%, 96%)
Luapula	20-49	89% (85%, 93%)	96% (92%, 99%)
Muchinga	0-4	77% (72%, 83%)	32% (25%, 39%)
Muchinga	5-9	80% (75%, 85%)	70% (64%, 76%)
Muchinga	10-14	83% (78%, 88%)	85% (81%, 90%)
Muchinga	15-19	90% (85%, 94%)	90% (85%, 94%)
Muchinga	20-49	94% (91%, 97%)	95% (91%, 99%)
Northern	0-4	71% (61%, 80%)	28% (19%, 37%)
Northern	5-9	88% (83%, 93%)	74% (67%, 81%)
Northern	10-14	84% (78%, 90%)	80% (73%, 86%)
Northern	15-19	90% (86%, 95%)	93% (89%, 97%)
Northern	20-49	96% (94%, 99%)	97% (95%, 100%)
North-Western	0-4	74% (68%, 81%)	29% (22%, 36%)
North-Western	5-9	91% (87%, 95%)	66% (60%, 73%)
North-Western	10-14	80% (75%, 86%)	82% (77%, 87%)
North-Western	15-19	89% (84%, 93%)	90% (86%, 95%)
North-Western	20-49	84% (77%, 91%)	93% (87%, 98%)

Southern	0-4	77% (71%, 83%)	24% (18%, 30%)
Southern	5-9	76% (71%, 82%)	61% (55%, 67%)
Southern	10-14	81% (76%, 86%)	79% (74%, 85%)
Southern	15-19	86% (81%, 91%)	87% (82%, 92%)
Southern	20-49	84% (77%, 90%)	96% (93%, 99%)
Western	0-4	73% (63%, 84%)	31% (20%, 42%)
Western	5-9	79% (72%, 86%)	56% (48%, 65%)
Western	10-14	87% (81%, 94%)	84% (76%, 91%)
Western	15-19	93% (89%, 97%)	87% (82%, 92%)
Western	20-49	86% (81%, 92%)	96% (93%, 99%)

Table S4: Measles and rubella survey weighted seroprevalence by age group (years) and province. See figures S1 and S7 for histograms of these numbers.

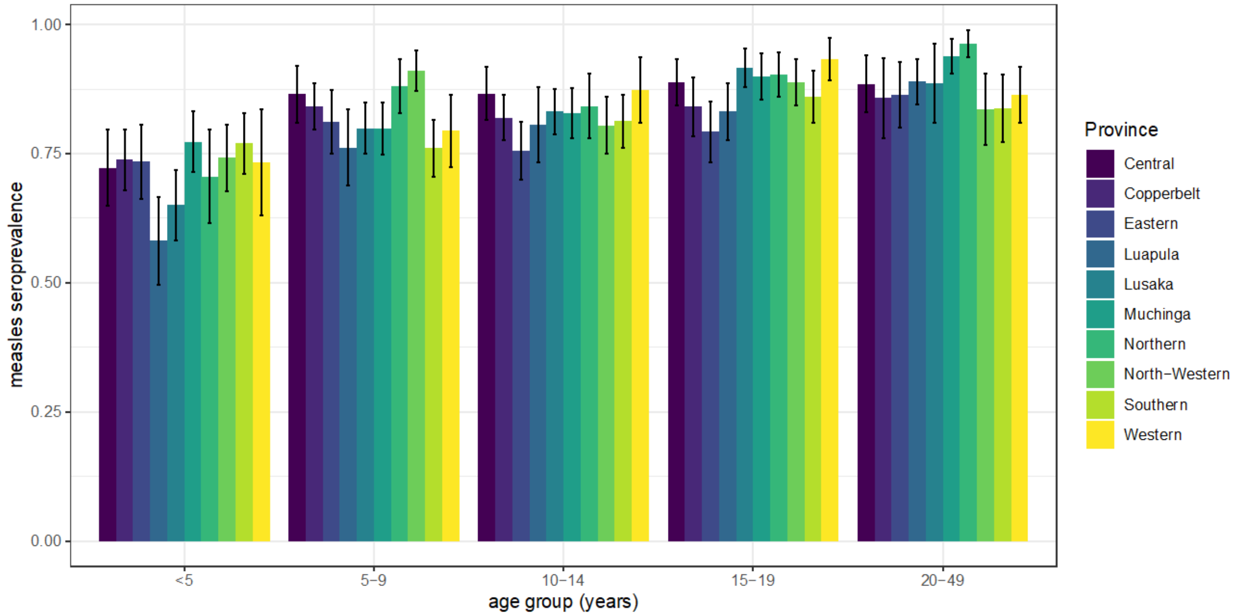

Figure S1: Measles survey weighted seroprevalence by age group and province

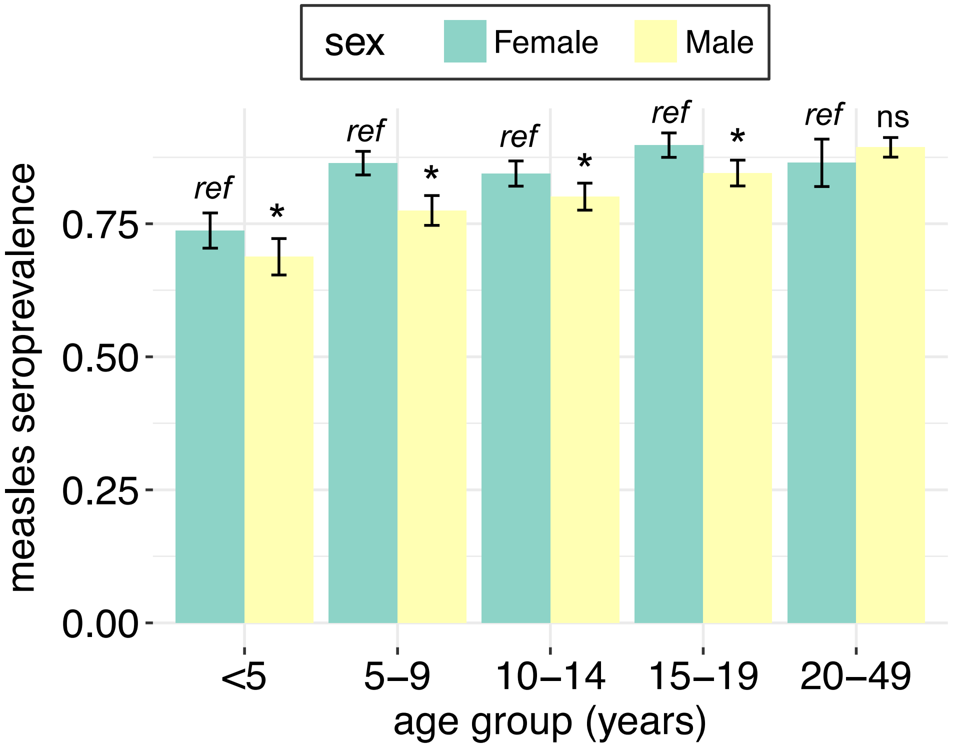

Figure S2: Measles survey weighted seroprevalence by age group and sex. Males had significantly lower measles seroprevalence than females in all age groups younger than 20 years; there were no significant differences in seroprevalence by sex in the 20-49 year age group. significance levels: *** 0.001; ** 0.01; * 0.05; ns not significant

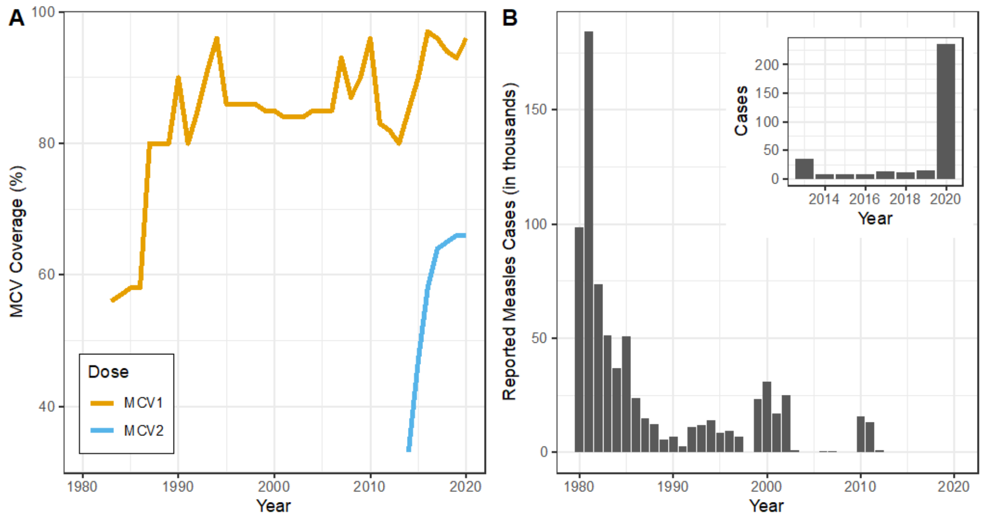

Figure S3: World Health Organization Zambia National Measles Epidemiologic Data. A) World Health Organization and United National Children's Fund (WUENIC) routine vaccination coverage estimates MCV1 and MCV2 in Zambia 1983-2020 and 2014-2020. B) Reported measles cases in Zambia 1980 to 2020.

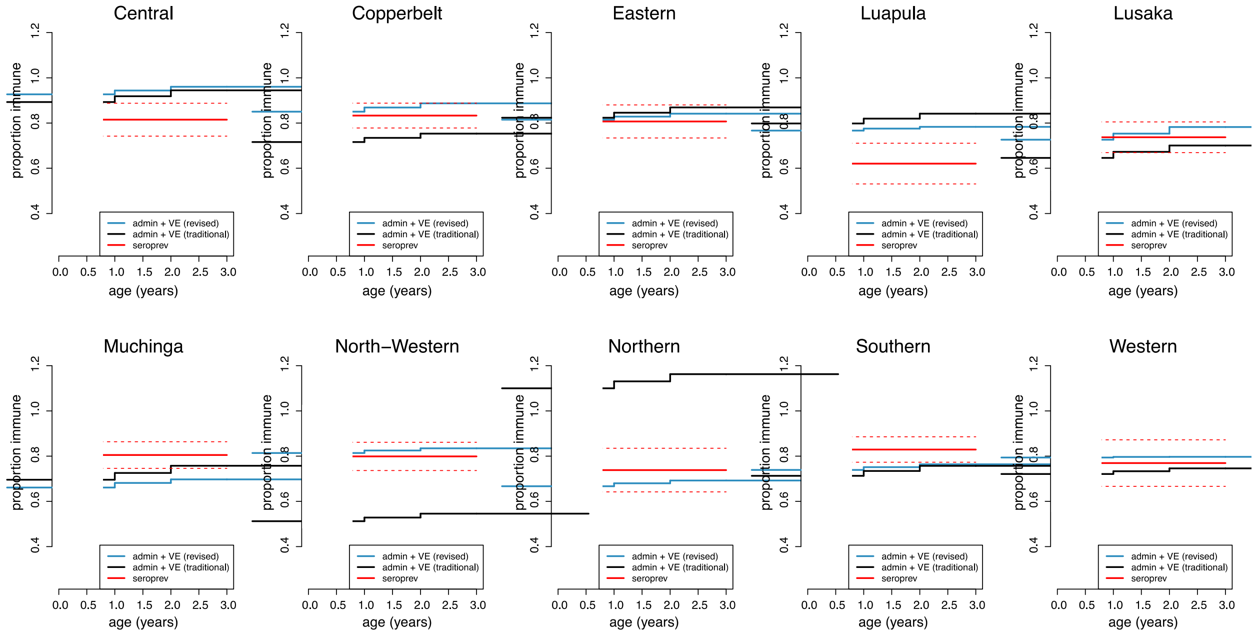

Figure S4: Estimated measles immunity by province in children 10 months to 4 years old. Indirect estimate of immunity based on MCV1 and MCV2 administrative (admin) coverage by year and province and vaccine efficacy (VE). The "revised" method (blue line) assumes that the population eligible for MCV1 vaccination is the number of individuals listed as age 0 in each province, and the population eligible for MCV2 vaccination is the number of individuals listed as age 1 in each province. The "traditional" method (black line) assumes that the population eligible for MCV1 vaccination is 4% of the total provincial population size, and the population eligible for MCV2 vaccination is 8% of the total provincial population size. Direct seroprevalence estimate as the survey weighted seroprevalence in the age group 10 months to 4 years (red line).

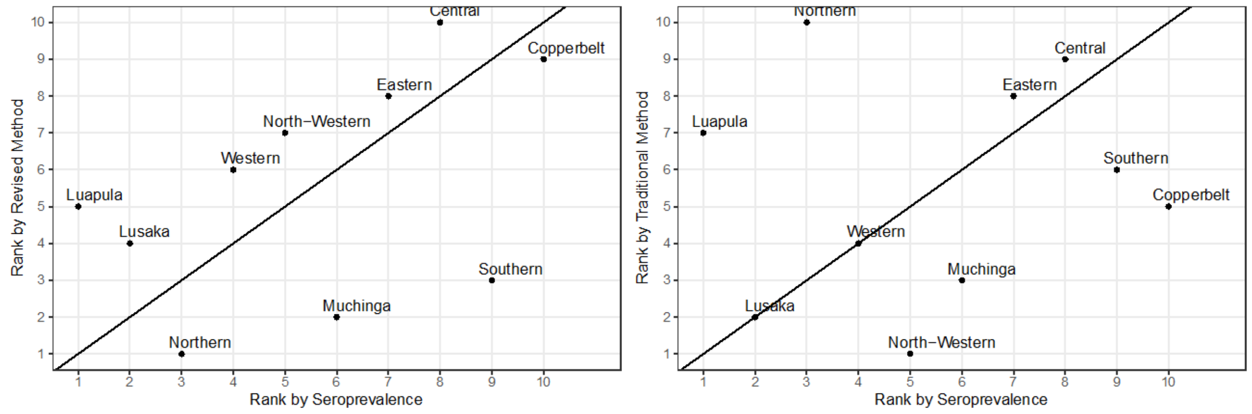

Figure S5: Comparison of provincial rank of estimated measles immunity in children 10 months to 4 years old for between seroprevalence and two different indirect estimates of immunity: "Revised Method" (left) and "Traditional Method" (right) (for further detail see Supplemental Methods or Figure S4). "Seroprevalence" is the survey weighted seroprevalence in the age group 10 months to 4 years. Each point represents a different province (10 total provinces) ranked from lowest (1) to highest (10) immunity estimate.

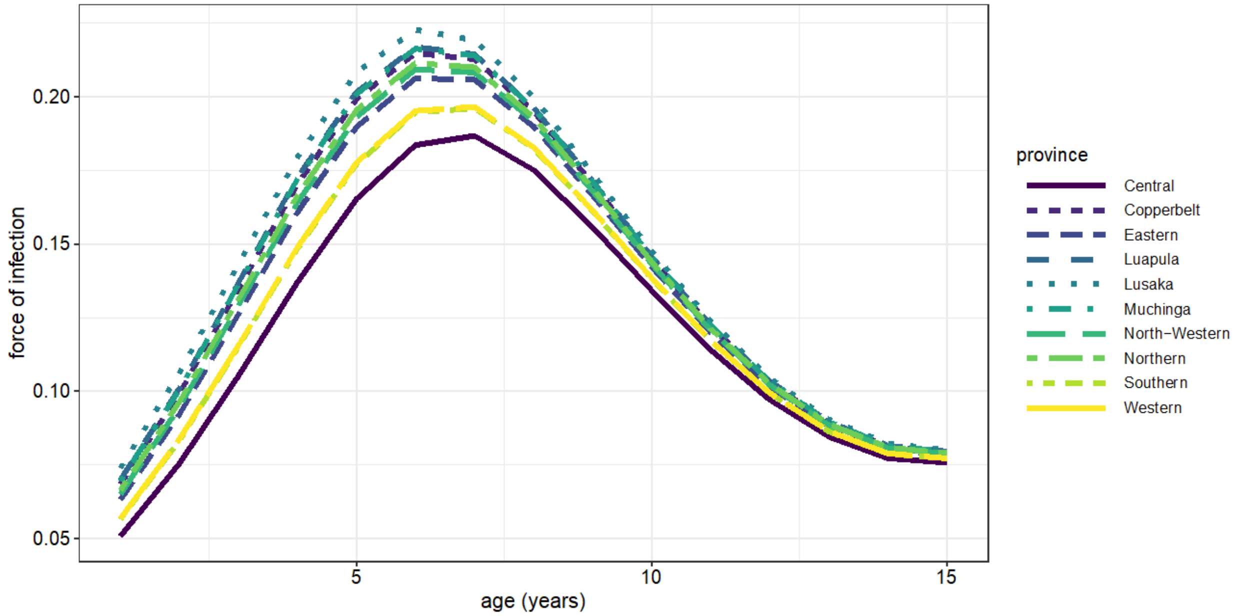

Figure S6: Rubella age-specific force of infection in children 1-15 years of age by province.

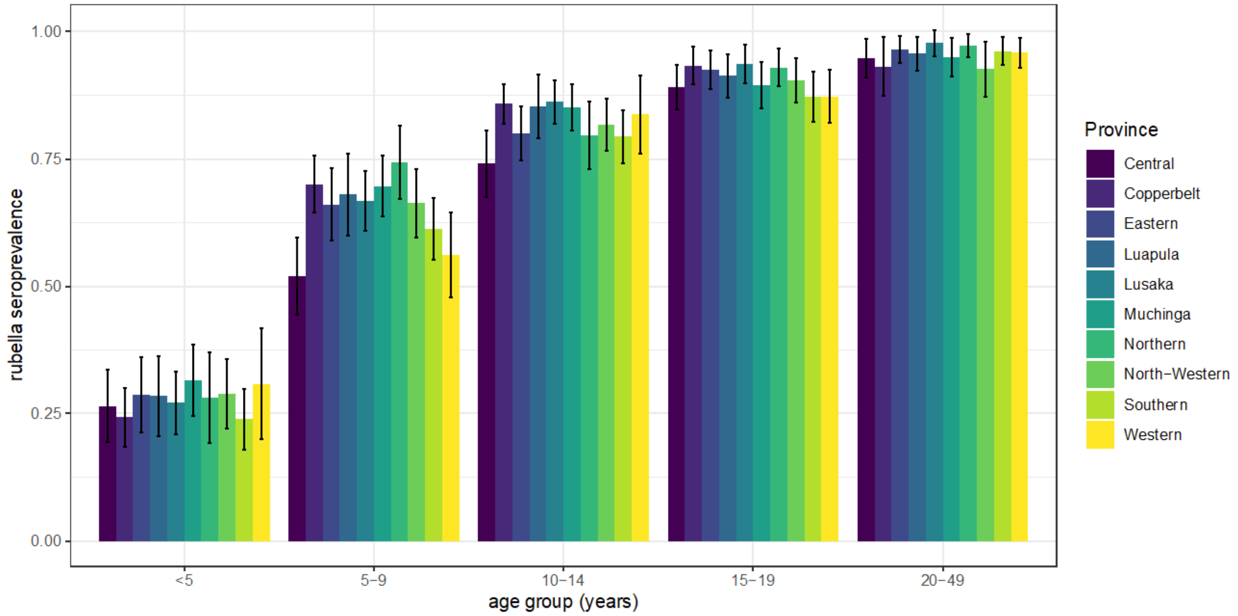

Figure S7: Rubella survey weighted seroprevalence by age group (years) and province

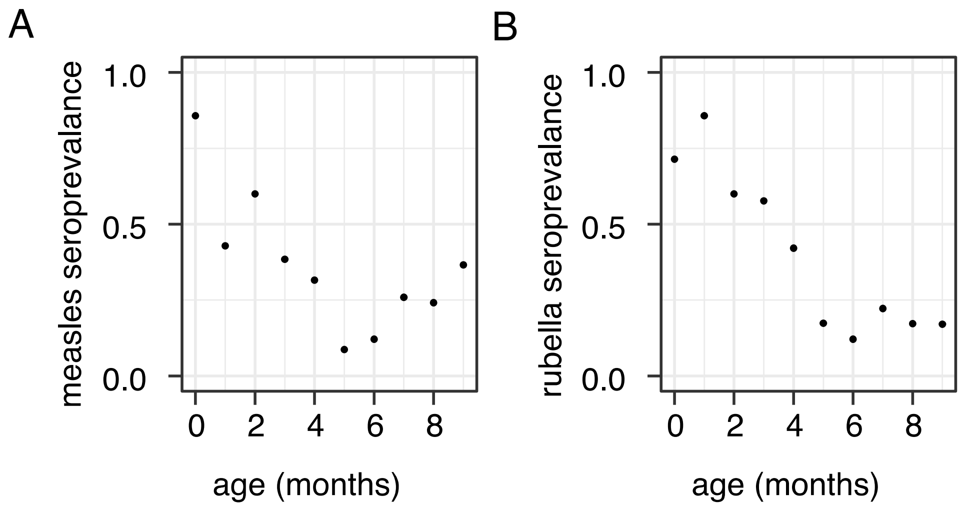

Figure S8: Measles (A) and rubella (B) seroprevalence 0 to 9 months old, grouped by ages in months. The number of samples for each age in months from 0 to 9 months were fairly small including 7, 21, 20, 26, 19, 23, 33, 27, 29, and 41 samples, respectively. Little inference can be made from these small samples, but there is a generally decreasing trend in measles seroprevalence between 0 and 5 or 6 months of age for both pathogens.

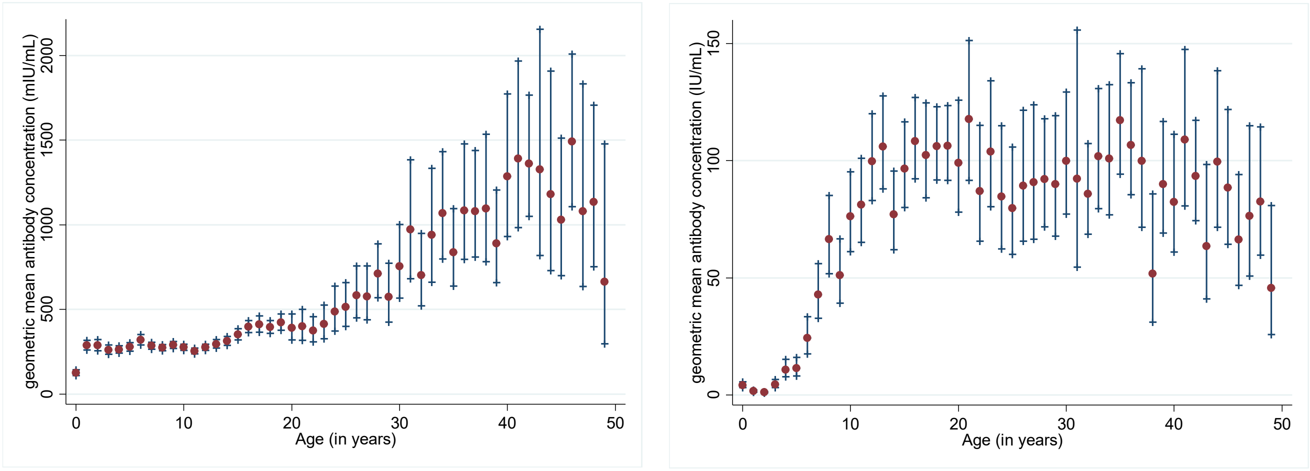

Figure S9: Measles (left) and rubella (right) geometric mean antibody concentrations and 95% confidence intervals, grouped by age in years. Measles antibody titers are somewhat flat from 1 to 20 years old, at which point concentrations and variation in concentrations start to increase. Rubella antibody titers and variation in concentration start to increase earlier in life around age 4 years.
